# Comprehensive *in vitro* characterization of PD-L1 small molecule inhibitors

**DOI:** 10.1038/s41598-019-48826-6

**Published:** 2019-08-27

**Authors:** Aravindhan Ganesan, Marawan Ahmed, Isobel Okoye, Elena Arutyunova, Dinesh Babu, William L. Turnbull, Joydeb Kumar Kundu, Justin Shields, Katharine Cheryl Agopsowicz, Lai Xu, Yasser Tabana, Nutan Srivastava, Guangzhi Zhang, Tae Chul Moon, Alexandr Belovodskiy, Mostofa Hena, Appan Srinivas Kandadai, Seyedeh Nargess Hosseini, Mary Hitt, John Walker, Michael Smylie, Frederick G. West, Arno G. Siraki, M. Joanne Lemieux, Shokrollah Elahi, James A. Nieman, D. Lorne Tyrrell, Michael Houghton, Khaled Barakat

**Affiliations:** 1 Faculty of Pharmacy and Pharmaceutical Sciences, University of Alberta, Edmonton, AB Canada; 2 Department of Dentistry, Faculty of Medicine & Dentistry, University of Alberta, Edmonton, AB Canada; 3 Department of Biochemistry, Faculty of Medicine & Dentistry, University of Alberta, Edmonton, AB Canada; 4 Department of Chemistry, Faculty of Science, University of Alberta, Edmonton, AB Canada; 5 Li Ka Shing Applied Virology Institute, University of Alberta, Edmonton, AB Canada; 6 Li Ka Shing Institute of Virology, University of Alberta, Edmonton, Alberta Canada; 7 Cancer Research Institute of Northern Alberta, University of Alberta, Edmonton, Alberta Canada; 8 Department of Oncology, Faculty of Medicine & Dentistry, University of Alberta, Edmonton, AB Canada; 9 Department of Medical Microbiology and Immunology, Faculty of Medicine & Dentistry, University of Alberta, Edmonton, AB Canada

**Keywords:** Cancer therapy, Immunotherapy

## Abstract

Blockade of the programmed cell death 1 (PD-1)/programmed cell death-ligand 1 (PD-L1) interaction has emerged as a powerful strategy in cancer immunotherapy. Recently, there have been enormous efforts to develop potent PD-1/PD-L1 inhibitors. In particular, Bristol-Myers Squibb (BMS) and Aurigene Discovery Technologies have individually disclosed several promising PD-1/PD-L1 inhibitors, whose detailed experimental data are not publicly disclosed. In this work, we report the rigorous and systematic *in vitro* characterization of a selected set of potent PD-1/PD-L1 macrocyclic peptide (BMSpep-57) and small-molecule inhibitors (BMS-103, BMS-142) from BMS and a peptidomimetic small-molecule inhibitor from Aurigene (Aurigene-1) using a series of biochemical and cell-based assays. Our results confirm that BMS-103 and BMS-142 are strongly active in biochemical assays; however, their acute cytotoxicity greatly compromised their immunological activity. On the other hand, Aurigene-1 did not show any activity in both biochemical and immunological assays. Furthermore, we also report the discovery of a small-molecule immune modulator, whose mode-of-action is not clear; however, it exhibits favorable drug-like properties and strong immunological activity. We hope that the results presented here will be useful in guiding the development of next-generation PD-1/PD-L1 small molecule inhibitors.

## Introduction

From the initial efforts of William Coley^1–3^, who demonstrated that treatment with live bacteria or bacterial products (Coley toxins) could regress tumors, in late 1800s to the recent Nobel Prize award to James Allison and Tasuku Honjo, for their discoveries of key immune checkpoints proteins^4^, cancer immunotherapy has come a long way. Today, immunotherapy has emerged as a powerful strategy for treating various types of cancers. This has become possible due to significant advances in our understanding about the molecular mechanisms behind the growth of cancers and their interplay with the host immune system, particularly the one mediated by T-lymphocytes (T cells).

T cells play a pivotal role in controlling the adaptive immune response against pathogens^5^ and in autoimmunity^6^. Upon activation, T cells mediate various cellular immune responses against pathogens such as chronic viral infections^7–11^. On the other hand, there is a class of negative immune modulatory molecules (called the immune checkpoints) that apply “brakes” to overactive immune responses to self-antigens by halting the T cell effector functions. This immune tolerance caused by immune checkpoints is crucial for establishing immunological equilibrium and preventing autoimmunity^12–14^. To date, several immune checkpoints have been identified, among which the programmed cell death 1 (PD-1) is a major negative regulator of T cell activation. PD-1 (or CD279) is a cell surface receptor that is expressed on activated T cells and it interacts with its related programmed cell death-ligands, PD-L1 and PD-L2, expressed on antigen presenting cells (APCs) (e.g., dendritic cells^15^). The surface recognition of PD-1 and PD-L1 (or PD-L2) transiently inhibits the effector T cells to maintain immune homeostasis^16–18^. However, cancer cells often exploit this adaptive immune resistance mechanism of the PD-1/PD-L1 pathway to their advantage and escape from the immunological surveillance of T cells. In this context, PD-L1 has been found to be overexpressed on the surface of different cancer types, including breast cancer, lung cancer, bladder cancer, lymphoma, glioblastoma and melanoma^19–23^. Thus, in the tumor microenvironment, the over-expressed PD-L1 binds to PD-1 and suppresses the T cell-mediated antitumor immune responses, thereby supporting survival and expansion of tumor cells. This has been confirmed by a number of studies based on mouse models^11,19,24,25^. Therefore, disrupting the protein-protein interactions of PD-1 and its ligands (PD-L1 and PD-L2) has become an attractive strategy for cancer immunotherapy.

The developments of Food and drug administration (FDA) approved PD-1-targeted monoclonal antibodies (mAbs) (*e*.*g*. nivolumab and pembrolizumab) and PD-L1 mAbs (*e*.*g*. atezolizumab, avelumab and durvalumab) have demonstrated the clinical effectiveness of targeting the PD-1/PD-L1 axis in different cancers^26–29^. Although these mAbs have transformed cancer immunotherapy forever, they still exhibit several disadvantages such as very long half-life, immune-related adverse effects (irAEs), low permeability, immunogenicity, complex production process, and exorbitant treatment costs^1,30–33^. Therefore, developing small-molecule inhibitors as an alternative to mAbs to block the PD-1/PD-L1 pathway (and other immune checkpoints) has emerged as an important area of drug discovery research.

The past few years witnessed a significant progress in designing PD-1/PD-L1 inhibitors^31,33–36^. In particular, Bristol-Myers Squibb (BMS)^37–40^ and Aurigene Discovery Technologies Limited^35,41–44^ have been leading in this avenue and have disclosed several PD-1/PD-L1 targeted inhibitors, including macrocyclic peptides, peptidomimetic molecules and non-peptidic small molecules. In particular, BMS revealed a number of thioether bond-containing macrocyclic inhibitors^38,39^ and small molecule inhibitors based on a 2-methyl-3-biphenyl-methanol scaffold^37^ with nanomolar range of activity (as determined by homogeneous time resolved fluorescence (HTRF) assay) for targeting the immunosuppressive PD-1/PD-L1 pathway^37,38^. Subsequent structural and biochemical studies showed that the macrocyclic inhibitors of BMS (BMS-57, BMS-71, and BMS-99) bind directly at the interface site of PD-L1 and block their interaction^45^. Nevertheless, the small-molecule inhibitors of BMS (BMS-8, BMS-200, BMS-202, BMS-242, BMS-1001, BMS-1166) have been shown to function through a novel dimer-locking mechanism, in which these molecules induce a dimerization of two free soluble PD-L1 proteins *via* their PD-1 binding epitopes^46,47^. This molecular packing induced by the small molecules makes it impossible for the recognition of PD-L1 by PD-1 *in vitro*. Aurigene Discovery Technologies also reported several peptidomimetic inhibitors that were developed from the sequences of the extracellular domain of PD-1 protein^35,41–44^. Most of these compounds were shown to rescue mouse splenocyte proliferation that was suppressed by human PD-L1. It should be noted that Aurigene recently collaborated with Curis Inc. to develop a small-molecule oral drug, CA-170, which is currently in Phase 1 clinical trials^48^. This CA-170, possibly comprises a 3-substituted-1,2,4 oxadiazole compound from their recent patent^43^, was claimed to induce effective proliferation and activation of T cells, which are suppressed by PD-L1 or VISTA. However, neither the structure nor any *in vitro* or *in vivo* data supporting the actual target, their cell-based activity or the abilities to release cytokines by this compound or any other Aurigene compounds has been reported in the literature. Despite the enormous attraction, the development of small-molecule inhibitors of the PD-1 pathway is clearly lagging behind mAb development. This is mainly due to the various challenges in developing drug-like small molecules that can occupy the shallow hydrophobic surfaces at the interface of these protein-protein interactions. Given such challenges, it is important to evaluate and understand the structure-activity-cytotoxicity relationships of these first generation PD-1/PD-L1 inhibitors, which can then guide the development of next generation compounds.

**Figure 1:**
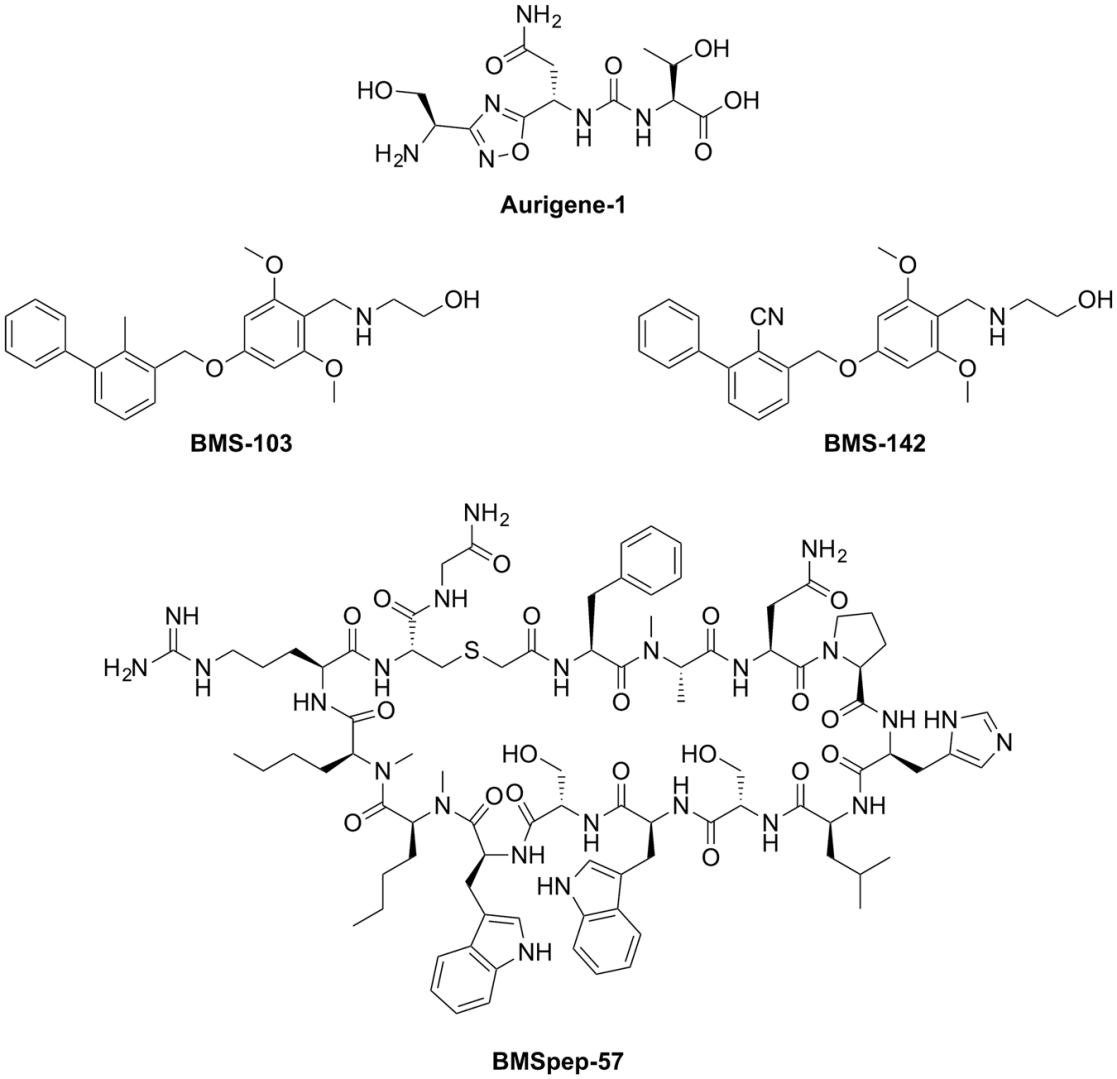
Chemical structures of PD-L1 inhibitors developed by Aurigene (Aurigene-1) and Bristol-Meyers Squibb (BMSpep-57, BMS-103, and BMS-142). Chemical structures were generated using ChemDraw Professional 15.

Toward this goal, we have carried out rigorous and systematic *in vitro* profiling of a selected set of promising inhibitors from both BMS and Aurigene (Fig. 1). Our choice of inhibitors covers three important categories: a macrocyclic peptide inhibitor (BMSpep-57)^38^, a peptidomimetic inhibitor (Aurigene-1)^43^, and non-peptidic small-molecule inhibitors (BMS-103, BMS-142)^37^. Compound BMSpep-57 was selected as a positive control since this compound has been extensively studied earlier through X-ray crystallography (*e*.*g*. Protein data bank (PDB) ID: 5O4Y)^45^, binding data (*e*.*g*. HTRF assay^38^, differential scanning fluorimetry (DSF) assay^45^, and Nuclear magnetic resonance (NMR)^45^ and cell-based PD-1/PD-L1 functional assay^45^. Compounds BMS-103 and BMS-142 were selected as they have been reported to be potent PD-1/PD-L1 blockers (IC_50_ in the range of 6–100 nM^37^), using a HTRF assay in the original disclosure^37^. However, no other experimental data are available for these compounds. Aurigene-1 was selected as it was also reported by Aurigene to have the best activity in terms of rescuing mouse splenocyte proliferation (97%) using a CFSE proliferation assay with recombinant PD-L1^43^. However, as indicated earlier, there is no additional experimental data supporting its binding to PD-L1 or its cell-based activity in the literature.

Here, we have employed a series of biochemical and cell-based assays along with modelling to evaluate the activity of these compounds towards the inhibition of the PD-1/PD-L1 interaction. Screening based on Differential scanning fluorimetry (DSF) assay, Microscale thermophoresis (MST), surface plasmon resonance (SPR) and NMR (for Aurigene-1 only) were performed to test the binding of these compounds to PD-L1. Their ability to interfere with the PD-1/PD-L1 interaction was validated by competitive ELISA assays. The cytotoxicity profiles of the compounds were also tested in Jurkat cells, CHO cells and human hepatic carcinoma cell line HepG2; and finally, the ability of the compounds to block PD-1/PD-L1 interaction and to improve interleukin-2 (IL-2) cytokine release was validated using a commercially available PD-1/PD-L1 cell-based assay and peripheral blood derived mononuclear cells (PBMCs) from healthy donors. We hope the extensive experimental data and analyses provided in this work can provide insight for designing the next generation PD-1/PD-L1 small molecule inhibitors.

## Results

### Detection of interaction of PD-L1 with compounds

Initially, we measured the binding properties of the selected BMS and Aurigene compounds towards the recombinant PD-L1 protein using three different *in vitro* assays, namely the DSF, MST, and SPR.

**Figure 2:**
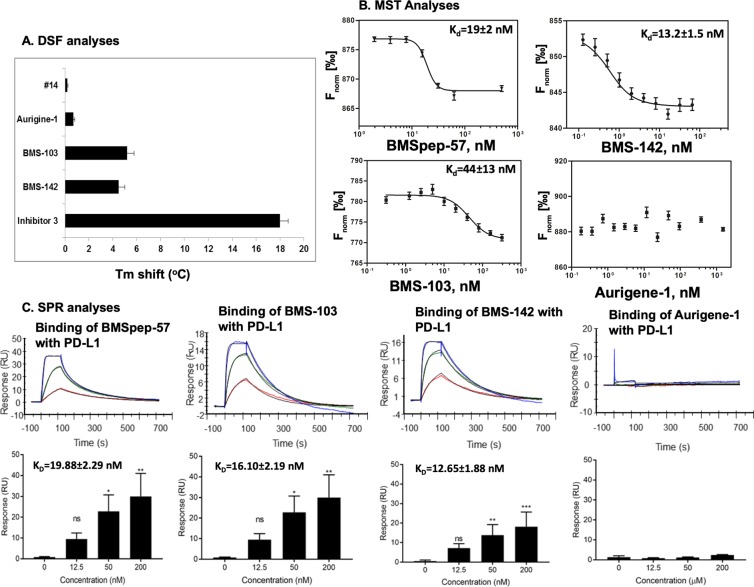
The binding of BMS molecules (BMSpep-57, BMS-103, BMS-142) and Aurigene-1 compound against human PD-L1 protein assessed using DSF (**A**), MST (**B**) and SPR (**C**) assays. (**A**) The shifts of T_m_ of PD-L1 in the presence of selected compounds. (**B**) The binding affinities of labeled PD-L1 to selected compounds as determined by MST. The difference in normalized fluorescence F_norm_ [‰] was plotted against MST T-Jump. Compound #14 was used as negative control and shows no binding to PD-L1. (**C**). The binding of the selected compounds with Fc-PD-L1 was evaluated using SPR technique. BMSpep-57, BMS-103, BMS-142 and Aurigene-1 compounds were allowed to flow over Fc-PD-L1 captured on a flow cell as well as on a reference cell of Series S Sensor Chip Protein A at indicated concentrations, and the R_max_ and K_D_ values were determined by Biacore Evaluation software. The solvent-corrected sensograms (representative ones on top panel) and the kinetic analysis of binding (bottom) were presented as mean ± SD from 3 to 4 independent assays (n = 4 for BMS-103 and BMS-142; n = 3 for BMSpep-57 and n = 2 for aurigene-1 binding with Fc-PD-L1).

The principle of DSF assay is based on the propensity of a protein to increase its thermal stability upon ligand binding. The thermal stability is defined by the melting temperature (T_m_). The magnitude of the T_m_ shift depends on many factors such as the concentration and affinity of the ligands, as well as the contributions of enthalpy and entropy of binding^49^. PD-L1 in the presence of 5% DMSO exhibited a melting temperature of 34.2 ± 0.2 °C, which is consistent with a T_m_ of 35.4 °C reported for PD-L1 by Skalniak *et al*.^40^. As expected, BMSpep-57, being a large macrocyclic peptide, demonstrated the biggest shift of T_m_ value of 18 ± 0.7 °C (Fig. 2A). In the presence of BMS-103 and BMS-142, the thermal stability of PD-L1 increased by 5.2 °C (T_m_ = 39.4 ± 0.4 °C) and 4.5 °C (T_m_ = 38.7 ± 0.3 °C), respectively (Fig. 2A). These data confirm the interactions of the BMS inhibitors with recombinant PD-L1. Although the T_m_ shift in the presence of BMSpep-57 may suggest the highest affinity towards PD-L1 in comparison with BMS-103 and BMS-142, these results should be interpreted with caution due to different physicochemical properties of compounds^50^. Interestingly, for Aurigene-1 the T_m_ shift was only 0.7 ± 0.1 °C (Fig. 2A), which indicates a lack of binding event between this compound and PD-L1. We also evaluated the binding of Aurigene-1 to the human PD-1 protein using DSF assay and found that the compound did not increase the thermal stability of PD-1 protein as well (data provided in Supplementary Fig. S1A). Thus, our DSF results suggest that Aurigene-1 binds neither to PD-1 nor to PD-L1. This is in agreement with an earlier study^47^, which reported that peptidomimetic agents from Aurgiene did not binding with either of the PD proteins; however no experimental data supporting this claim was disclosed.

Next, we employed the MST technique to evaluate the binding of the BMS and Aurigene compounds with PD-L1 and also to determine their binding constants (K_d_) (Fig. 2B). MST enables the direct monitoring of the protein-compound complex formation, given that one partner carries a fluorescent label^51^. In the course of MST experiments, the concentration of labeled PD-L1 was kept constant at 60 nM, while the concentrations of the compounds were varied. After a short incubation, the samples were loaded into standard treated glass capillaries and MST analysis was performed. The complex formation led to a fluorescent signal that enabled the measurement of affinities (K_d_). Binding of all three tested BMS compounds (BMS-103, BMS-142 and BMSpep-57) to labeled PD-L1 resulted in a clear response in fluorescence signal that was dependent on compound concentration. The calculated K_d_ values for the interaction of BMS-103 and BMS-142 with PD-L1 were 44 ± 13 nM and 13.2 ± 1.5 nM, respectively, when compared to K_d_ of 19 ± 2 nM for BMSpep-57 (Fig. 2B). In contrast to the BMS inhibitors, the treatment with different concentration of Aurigene-1 showed no change in fluorescence (Fig. 2B), which is similar to the behavior of our negative control (compound #14 in Supplementary Fig. S1B) in this study. This suggests that Aurigene-1 does not bind to PD-L1, which is consistent with the finding from DSF.

We also evaluated the kinetics of binding of the compounds to PD-L1 using SPR (Fig. 2C). SPR is a sensitive and a label-free biophysical method to study protein-protein and small molecule-protein interactions. Recombinant Fc-PD-L1 (5 μg/ml) protein was captured on a Series S Sensor Chip Protein A at a level of ~1000 RU as the ligand. Initial binding kinetics analysis of PD-1/PD-L1 interactions was performed by flowing His-tagged PD-1 (at concentrations of 0, 0.12825, 0.513 and 2.052 μM) over the Fc-PD-L1-captured sensor chip, which resulted in a maximum binding response (R_max_) of 143.3 ± 9.47 RU and the calculated K_D_ value of 1.2 ± 0.25 μM (Supplementary Fig. S2). Later, the interactions of the BMS inhibitors (BMSpep-57, BMS-103, BMS-142) and Aurigene-1 compound with PD-L1 were measured by allowing the compounds to flow over the captured Fc-PD-L1 in one flow cell as well as in a reference flow cell having no captured ligand (Fig. 2C). Analyses of the resulting binding kinetics revealed that BMSpep-57 bound with Fc-PD-L1 with an R_max_ value of 30.58 ± 6.24 RU and a calculated K_D_ value of 19.88 ± 2.29 nM. BMS-103 interacted with Fc-PD-L1 with R_max_ of 18.66 ± 3.55 RU; whereas BMS-142 bound Fc-PD-L1 with 18.14 ± 2.48 RU (Fig. 2C). The K_D_ values for the binding of BMS-103 and BMS-142 with Fc-PD-L1 were 16.10 ± 2.19 nM and 12.64 ± 1.88 nM, respectively. To verify whether the binding of BMSpep-57, BMS-103 and BMS-142 with Fc-PD-L1 is specific, we examined the binding of these inhibitors with Fc-B7-1, another IgV-based immune checkpoint ligand that binds with CTLA-4 and CD28 receptors. None of the BMS inhibitors (BMSpep-57, BMS-103 and BMS-142) showed binding with the captured Fc-B7-1 on the sensor chip (Supplementary Fig. S2). However, the endogenous B7-1 binding partner CTLA-4 bound with Fc-B7-1, as expected, with a R_max_ value of 85.98 RU and K_D_ value of 7.37 nM, (Supplementary Fig. S2) which is consistent with previously reported K_D_ value (9.58 nM) of B7.1 interaction with CTLA-4^52^. The Aurigene-1 compound, however, showed a very small R_max_ value of 0.78 ± 0.36 RU. Therefore, SPR data concurs with our results from DSF assay and MST that Aurigene-1 compound does not bind with PD-L1.

In addition, we also employed 1D NMR-based methods, saturation transfer difference (STD) and WaterLOGSY techniques, to unambiguously rule-out the binding of Aurigene-1 to PD-L1 protein. STD and WaterLOGSY are popular ligand-based approaches to detect the binding of protein-protein inhibitors^53^. Our NMR data (provided in Supplementary Fig. S3) also confirm that Aurigene-1 does not bind to PD-L1 protein.

### Abilities of compounds to block PD-L1/PD-1 interactions in competition ELISA

**Figure 3:**
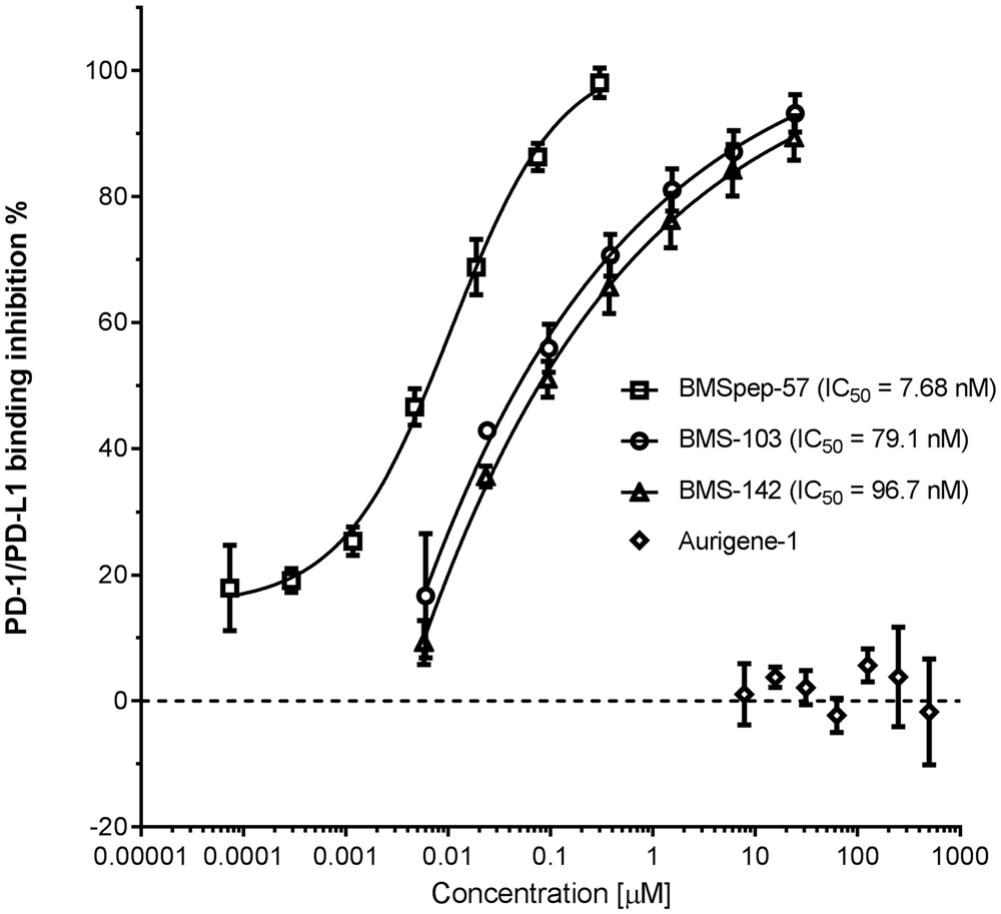
Ability of compounds to inhibit PD-1/PD-L1 binding in an ELISA competition assay. Compounds were pre-incubated in plates coated with PD-L1 followed by incubation with biotinylated PD-1. The PD-1/PD-L1 binding inhibition percentage was calculated from the difference in the amount of biotinylated PD-1 bound in the presence or absence (vehicle alone) of compound. In the absence of a compound, binding inhibition was normalized to 0% (dotted line). Data represent mean ± SD from three independent experiments, each independent experiment was run with three technical replicates. Where possible, curves were fitted using a 4PL function and the IC_50_ value was determined by interpolation.

To test whether the tested compounds were able to inhibit PD-1/PD-L1 binding, we established a binding inhibition assay using purified proteins (Fig. 3). In the presence or absence of a compound, we measured the amount of biotin tagged PD-1 that was able to bind to the wells coated with PD-L1. Initially and as a proof of principle, we showed that an anti-PD-1 mAb completely inhibited the PD-1/PD-L1 association (data shown in Supplementary Fig. S4). Using this assay, we showed that BMSpep-57 was able to inhibit PD-1/PD-L1 binding up to 98.1% (±2.36 SD) at 300 nM. In addition, across a four-fold serial dilution (300 nM to 0.073 nM), BMSpep-57 showed a concentration dependent inhibition of PD-1/PD-L1 binding with an IC_50_ of 7.68 nM. The compounds BMS-103 and BMS-142 were also tested across a four-fold serial dilution (24 µM to 0.006 µM) and also demonstrated concentration dependent inhibition of PD-1/PD-L1. BMS-103 showed up to 93.3% (±2.00% SD) inhibition at 24 µM and an IC_50_ of 79.1 nM. BMS-142 showed similar inhibition of up to 89.3% (±3.53% SD) at 24 µM and an IC_50_ of 96.7 nM.

In contrast to the BMS inhibitors, Aurigene-1 did not inhibit the PD-1/PD-L1 binding in any of the tested concentrations, ranging from 7.8 µM to 500 µM (Fig. 3). To check if the inability of Aurigene-1 to inhibit binding was due to the immobilization of PD-L1 in this assay, we performed the assay in the reverse configuration: with biotin tagged PD-L1 added to wells coated with PD-1 (Supplementary Fig. S5). The functionality of this assay was also verified using the same anti-PD-1 mAb, which completely inhibited the PD-1/PD-L1 interaction. Nevertheless, Aurigene-1 still did not show inhibition of PD-1/PD-L1 binding.

### Interleukin-2 (IL-2) and NFAT luciferase reporter activity of the compounds

**Figure 4:**
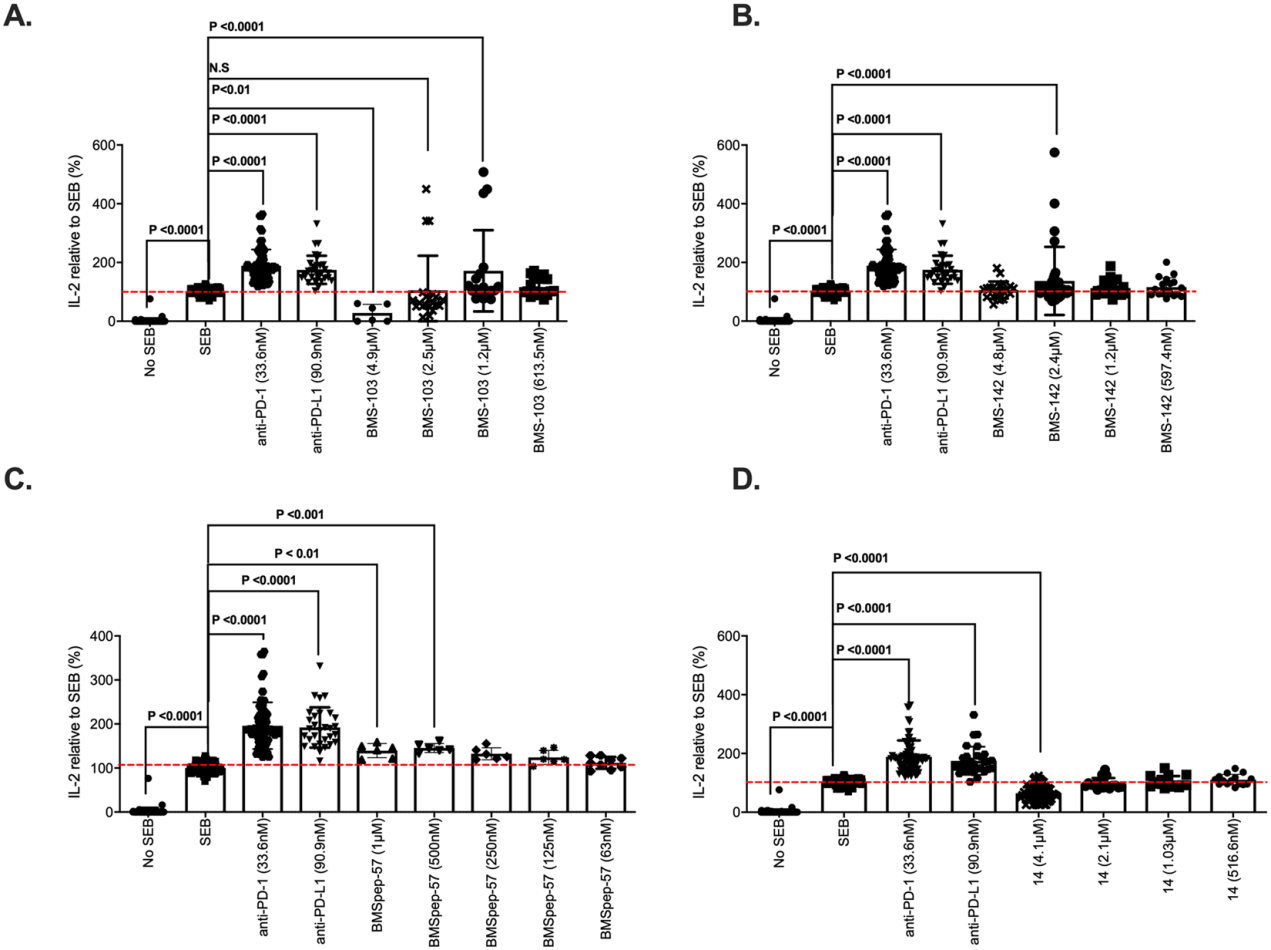
Fold IL-2 production by SEB-stimulated peripheral blood mononuclear cells pre-treated with monoclonal antibodies against PD-1 (33.6 nM), PD-L1 (90.9 nM) or the indicated BMS compounds relative to the vehicle treatment. (**A**) Effect of BMS-103 on IL-2 production of PBMC. (**B**) Effect of BMS-142 on IL-2 production of PBMC. (**C**) Effect of BMSpep-57 on IL-2 production of PBMC. (**D**) Effect of compound 14, a negative control used in this study on IL-2 production by PBMCs. Cumulative data showing mean ± SD from five experiments (BMS compounds) shown. P-values indicate statistical significance calculated using the Kruskal-Wallis test followed by Dunn’s multiple comparisons test. Unstimulated (No SEB) cells and untreated stimulated cells (SEB) were used as negative and positive controls, respectively.

In order to evaluate immunological activity of the tested compounds, we first investigated their ability to promote T cell function. We analyzed the production of IL-2 by peripheral blood mononuclear cells (PBMCs) pre-treated with PD-1/PD-L1 antagonists (or inhibitors): neutralizing mAbs or two-fold concentrations of BMS compounds (*i*.*e*. BMS-103 and BMS-142) before stimulation with Staphylococcal enterotoxin B (SEB) for 72 h (Fig. 4). We utilised this *in vitro* system as PBMCs consist of cells that express/up-regulate both PD-1 (T cells) and PD-L1 (T cells, APCs) upon stimulation. Cytokine levels from cell culture supernatants indicate that as expected, stimulated T cells treated with a-PD-1/PD-L1 neutralizing mAb produced significantly higher concentrations of IL-2 compared to untreated and stimulated cells (2- to 4-fold higher, P < 0.0001, Fig. 4). On the other hand, the levels of IL-2 induced by BMS compounds investigated varied (Fig. 4). We observed that T cells treated with a 1.2 μM concentration of BMS-103 (Fig. 4A) and 2.4 μM BMS-142 (Fig. 4B) elicited significantly higher levels of IL-2 than the “SEB-only” positive control (2- to 5-fold higher, P < 0.0001 Fig. 4), while the PD-1/L1 inhibitor, BMSpep-57 induced high levels of IL-2 at 1 µM and 500 nM concentrations (~1.5-fold; P < 0.01, Fig. 4C). Other concentrations investigated did not impact IL-2 production; our observations imply that a high concentration of compounds maybe toxic for T cells. For instance, T cells treated with a 4.9 μM concentration of BMS-103 produced significantly lower levels of IL-2 than the positive control and similar levels to the “No SEB” negative control (P < 0.01 and n.s, respectively, Fig. 4). We have evaluated the cytotoxicity of the studied compounds, which are discussed in the latter section. As expected the negative control compound #14 failed to impact IL-2 production in SEB-stimulated PBMCs (Fig. 4D).

**Figure 5:**
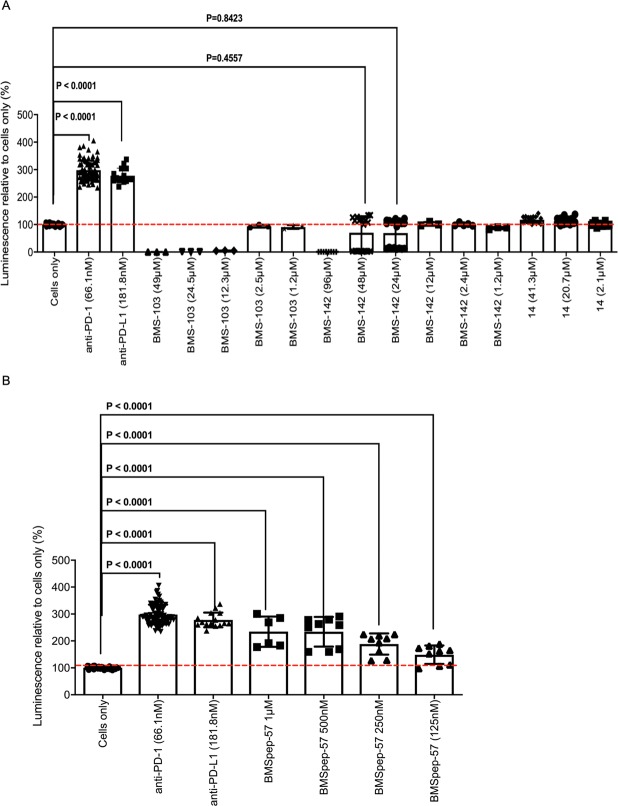
NFAT-luciferase reporter activity elicited by PD-1/NFAT Reporter-Jurkat cells treated with neutralizing antibodies (PD-1 or PD-L1) or BMS compounds after co-culture with TCR activator/PD-L1-CHO cells. Fold luminescence of Jurkat cells treated with antibodies (α-PD-1 (66.1 nM), α-PD-L1; (181.8 nM)) or the indicated BMS compounds (BMS-103, BMS-142, BMSpep-57, and negative control compound #14) compared to the negative control (cells only). P-values indicate statistical significance calculated using the Kruskal-Wallis test followed by Dunn’s multiple comparisons test or Mann-Whitney non-parametric test. Untreated (cells only) and mAb-treated (PD-1 or PD-L1) Jurkat cells served as negative and positive controls, respectively. Cumulative data showing mean ± SD from ten experiments shown.

Treatment of PBMCs with BMS compounds and concomitant production of IL-2 implies their ability to promote T cell responses, which is suggestive of interaction with PD-1/PD-L1. In this regard, to confirm whether the modest increase in IL-2 production by T cells treated with BMS compounds was due to inhibition of PD-1/PD-L1 signaling, we tested if these compounds can disrupt PD-1/PD-L1 interaction in a bioluminescent cell-based assay. Luminescence corresponding to nuclear factor of activated T cells (NFAT) activity by PD-1/NFAT reporter Jurkat T cells treated with anti-PD-1 and PD-L1 neutralizing antibodies were 3 to 4-fold higher than untreated cells as expected (P < 0.0001, Fig. 5A). Similar to our observations for IL-2 production, responses induced by BMS compounds varied, compared to the “cells only” negative control. This ranged from no change in NFAT activity (BMS-103 (2.5 μM and 1.2 μM), BMS-142 (12.3 μM, 2.4 μM and 1.2 μM)), to significantly reduced activity (BMS-103 (49 μM and 24.5 μM)) (Fig. 5A). These observations suggest that although lower concentrations of these BMS compounds may elicit IL-2 production by T cells, they are insufficient to compromise PD-1/PD-L1 interaction and hence NFAT activity.

We found that NFAT activity induced by the PD-1/PD-L1 macrocyclic inhibitor, BMSpep-57, correlated with their ability to induce IL-2 production in T cells (Fig. 5B). The luminescence of PD-1/NFAT reporter Jurkat T cells pre-treated with 1 μM and 500 nM BMSpep-57 were ~3-fold higher than the “cells only” negative control (P < 0.0001, Fig. 5B). Furthermore, lower concentrations of BMSpep-57 (250 nM and 125 nM) induced significantly higher NFAT activity in pre-treated Jurkat T cells (~2-fold, P < 0.0001, Fig. 5B). From these observations we can deduce that BMSpep-57 compromises PD-1/PD-L1 interaction at lower concentrations than correlated with increased IL-2 production.

To confirm whether our observations were dependent on interaction with PD-1, we investigated NFAT activity by treated PD-1 negative Jurkat T cells. We found no difference between the luminescence of PD-1 negative Jurkat cells left untreated or treated with anti-PD-1 (Supplementary Figs S6, 7). Furthermore, the luminescence of PD-1 negative Jurkat T cells treated with concentrations of BMS-142 that elicited NFAT activity in PD-1-expressing Jurkat cells were similar to background levels. Also, the luminescence of PD-1 negative Jurkat cells treated with 24.5 μM and 49 μM of BMS-103 were substantially low (P < 0.000, Supplementary Fig. S6) compared to background and Jurkat cells exposed to other conditions, indicating toxicity at these concentrations as seen with treated PD-1+ Jurkat cells.

**Figure 6:**
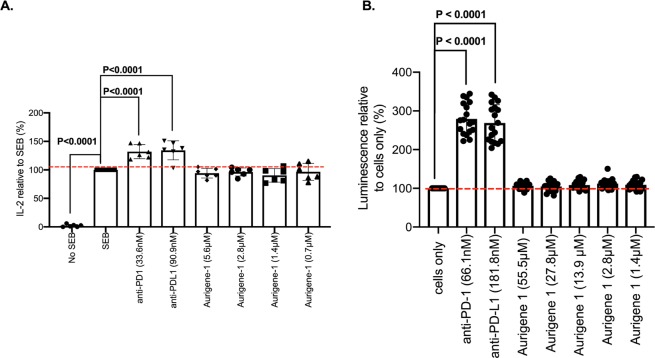
Cell-based activity of Aurigene-1 compound. (**A**) Fold IL-2 production by SEB-stimulated peripheral blood mononuclear cells pre-treated with monoclonal antibodies against PD-1 (33.6 nM), PD-L1 (90.9 nM) or two-fold concentrations of Aurigene-1 compared to the positive control (SEB). P-values indicate statistical significance calculated using the Kruskal-Wallis test followed by Dunn’s multiple comparisons test. Unstimulated (No SEB) cells and untreated stimulated cells (SEB) were used as negative and positive controls, respectively. Data from two experiments using three biological replicates shown. Unstimulated (No SEB) cells and untreated stimulated cells (SEB) were used as negative and positive controls respectively. (**B**) NFAT-luciferase reporter activity elicited by PD-1/NFAT Reporter-Jurkat cells treated with neutralizing antibodies (PD-1 or PD-L1) or Aurigene-1. Fold luminescence of Jurkat cells treated with antibodies (a-PD-1, 66.1 nM and a-PD-L1, 181.8 nM) or two-fold concentrations of the indicated Aurigene compounds compared to the negative control (cells only). P-values indicate statistical significance calculated using the Kruskal-Wallis test followed by Dunn’s multiple comparisons test. Untreated (cells only) and mAb-treated (PD-1 or PD-L1) Jurkat cells served as negative and positive controls, respectively. Cumulative data showing mean ± SD from five experiments shown.

We also explored if the Aurigene-1 small-molecule can facilitate IL-2 production in response to SEB stimulation and also interfere with PD-1/PD-L1 interaction. Our results show that Aurigene-1 cannot enhance IL-2 production by treated PBMCs at all concentrations investigated (5.6 μM, 2.8 μM, 1.4 μM and 0.7 μM, Fig. 6A). Unlike mAb-treated PBMCs, IL-2 levels elicited in response to Aurigene-1 were comparable to the “SEB only” (Fig. 6A). Regarding the bioluminescence assay to confirm interference with PD-1/PD-L1 interaction we treated PD-1/NFAT reporter Jurkat T cells with both high and low concentrations of this compound (55.5 μM, 27.8 μM, 13.9 μM, 2.8 μM and 1.4 μM, Fig. 6B). Similar to our observations from the IL-2 ELISA, none of these concentrations facilitated NFAT activity by treated Jurkat cells. These results indicate that Aurigene-1 is not able to promote IL-2 production and possibly other T cell functions in our PD-1/PD-L1 cell-based assays.

### Evaluation of cytotoxicity of the compounds in Jurkat, CHO and HepG2 cells

**Figure 7:**
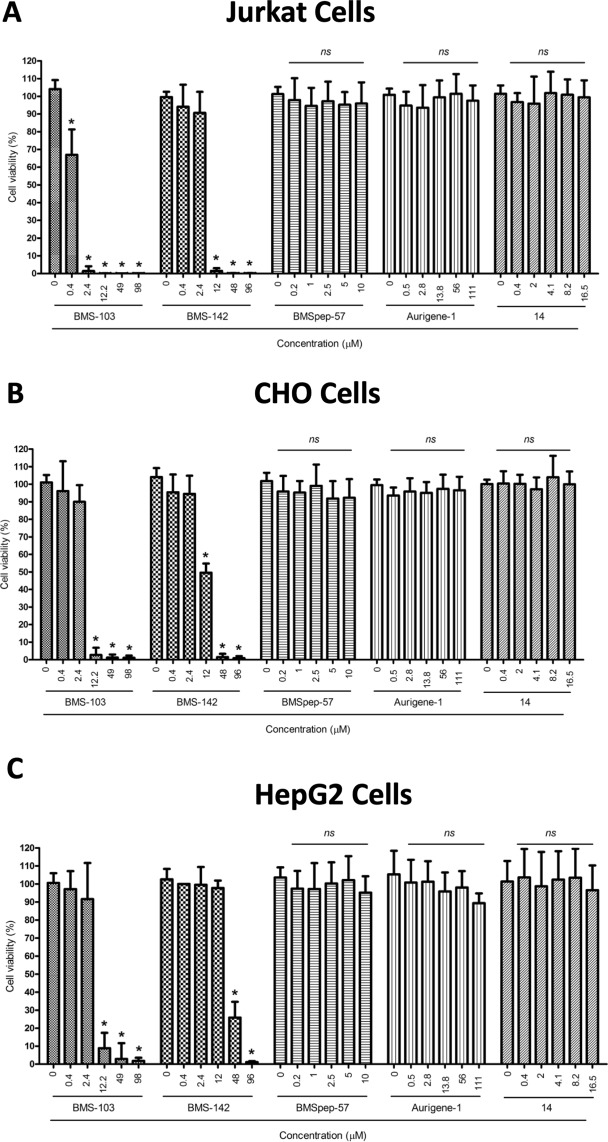
Concentration-dependent effects on viability of the indicated test compounds on Jurkat, CHO and HepG2 cells. Jurkat (**A**), CHO (**B**) or HepG2 (**C**) cells were treated with various concentrations of different test compounds (BMS-103, BMS-142, BMSpep-57, Aurigene-1 and compound #14) for 24 h, and the cell viability was assessed by the CellTiter-Glo^®^ Luminescent Cell Viability Assay (**A**,**B**) and the CCK-8 assay (**C**). Cumulative data from three independent experiments are shown. *ns* = non-significant in comparison with control (DMSO-treated cells). *P < 0.0001 indicates statistical significance calculated by using one-way ANOVA followed by Dunnett’s multiple comparison’s test.

In order to evaluate whether the tested compounds can affect the viability of both Jurkat and CHO cells that were used in bioluminescent cell-based assay for PD-1/PD-L1 inhibitor screening, cells were treated with the compounds for 24 h and then the cell viability was assessed by CellTiter-Glo^®^ Luminescent Cell Viability Assay (Fig. 7A,B). In the case of Jurkat cells, BMS-103 significantly  decreased cell viability at 0.4 µM compared to the DMSO-treated control and completely abolished the cell viability with concentrations at 2.4 µM and greater (*P* < 0.0001, Fig. 7A; grid bars). BMS-142 did not show any effect on cell viability up to 2.4 µM whereas the viability was significantly reduced from 12 µM and greater (*P* < 0.0001, Fig. 7A; checked bars). BMSpep-57, Aurigene-1 and the negative control compound #14 did not show any effect on the cell viability at the concentrations tested (*ns*, Fig. 7A; vertical, horizontal and crossbars, respectively).

In the case of CHO cells, BMS-103 significantly reduced the cell viability beginning from 12.2 µM and greater as compared to the DMSO-treated control group (*P* < 0.0001, Fig. 7B; grid bars). BMS-142 did not show any effect on CHO cell viability up to 2.4 µM whereas the viability was significantly reduced beginning from 12 µM and greater as compared to the control group (*P* < 0.0001, Fig. 7B; checked bars). These results correlate with our cell-based data showing that the BMS small molecules become significantly toxic at higher concentrations (Figs 4 and 5). Similar to their effects on Jurkat cells, BMSpep-57, Aurigene-1 and the negative control compound #14 did not show any effect on the CHO cell viability at the various concentrations tested (*ns*, Fig. 7B; vertical, horizontal and crossbars, respectively).

We also evaluated the acute cytotoxicity of the BMS and Aurigene compounds by testing the effects of compound treatment on the viability of HepG2 cell line. Metabolism, although less active compared to primary hepatocytes, may lead to metabolites, some of which may be cytotoxic. The purpose of using HepG2 cells, thus, would allow for evaluation of the parent molecules and potential metabolites for cytotoxicity. The cells were treated with the compounds for 24 h and then the cell viability was assessed by the CCK-8 assay (Fig. 7C). BMS-103 did not show any effect on cell viability up to 2.4 µM compared to the DMSO-treated control group; whereas, the viability was significantly reduced from 12.2 µM in a concentration-dependent manner (*P* < 0.0001, Fig. 7C). BMS-142 showed a significant reduction in cell viability at 48 µM and higher concentrations compared to the control group (*P* < 0.0001, Fig. 7C). On the other hand, the macrocyclic inhibitor, BMSpep-57, and Aurigene-1 compound did not show any effects on the cell viability at the various concentrations tested (Fig. 7C).

### Molecular modeling of PD-L1 binding with small molecules

The crystal structure of BMSpep-57/PD-L1 complex (PDB: 5O4Y)^45^ has been reported previously. BMSpep-57 is a 15-amino acid-containing cyclic peptide that is found to bind at the GFCC’ side of PD-L1, which is the actual binding face for PD-1 protein (Supplementary Fig. S8). The complex of BMSpep-57/PD-L1 is mainly stabilized by a number of hydrophobic interactions that are also seen within the complexes of mAbs and PD-L1^45^. Thus, given its large size and the target-binding site, BMSpep-57 is able to effectively target the PD-1/PD-L1 axis, as seen by its superior activity in the biochemical and cell-based assays.

**Figure 8:**
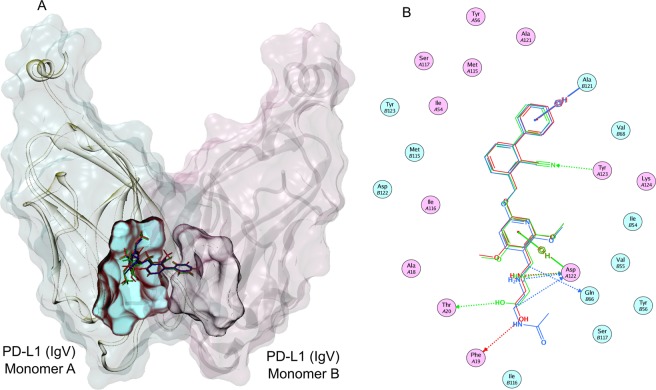
Comparison of the binding modes of BMS-103, BMS-142, and the reference BMS-202 compound against a PD-L1 dimer at molecular-level. (**A**) The superposed binding modes of BMS-103 (red stick) and BMS-142 (green stick), along with the reference BMS-202 compound (blue stick), within an induced dimer of two PD-L1 IgV domains. The two monomers of PD-L1 IgV domain are shown in cartoon representations. The surface of monomer A of PD-L1 is shown in cyan; whereas, the surface of monomer B of PD-L1 is shown in pink. The binding sites for BMS small molecules on both the monomers are also mapped in corresponding colors (cyan for monomer **A**; pink for monomer **B**). (**B**) A closer view of the binding-modes and interactions of the BMS small molecules sandwiched between the two PD-L1 monomers, leading to a stable PD-L1 dimer. The key hydrogen bond interactions and arene-pi interactions are also shown.

Previous X-ray crystal studies^46,47^ revealed an unexpected mechanism in which the selected BMS small-molecule inhibitors from 2-methyl-3-biphyenyl-methanol scaffold not only bind specifically to PD-L1; but also induce dimerization of soluble PD-L1 thereby obstructing the PD-1/PD-L1 axis. However, the 3D models representing the binding of BMS-103 and BMS-142 with PD-L1 are not available. Therefore, we modeled their 3D complex structures using pharmacophore-based docking approach based on the homologous crystal structure of BMS-202/PD-L1 complex (PDB: 5J89)^47^, followed by short molecular dynamics (MD) refinements (see methods). Our models suggested that, despite some structural changes on the biphenyl ring of BMS small molecules, the presence of three common aromatic pharmacophore features could allow these compounds to bind in the same way as BMS-202 and induce PD-L1 dimerization (Fig. 8). Our binding affinity calculations demonstrated that the BMS small molecules were more stable when bound within a dimer configuration (relative ΔG > 50 kcal mol^−1^) than to that of a PD-L1 monomer (relative ΔG < 25 kcal mol^−1^). When sandwiched within an induced PD-L1 dimer, the BMS small molecules were able to bury within the hydrophobic patches that come from the two monomers, especially from Tyr56, Met116 and Ala121. In particular, the compounds lock the CC’ strands from the two PD-L1 monomers to cover the binding site (Fig. 8). In this proposed binding pose, the BMS small molecules also formed H-bond interactions with Asp122 and Gln66 that stabilize the complex. As demonstrated by the results from our cell-based assays, these BMS small molecules interlocking two PD-L1 monomers are also able to induce T cell activity although they become toxic at higher concentrations.

**Figure 9:**
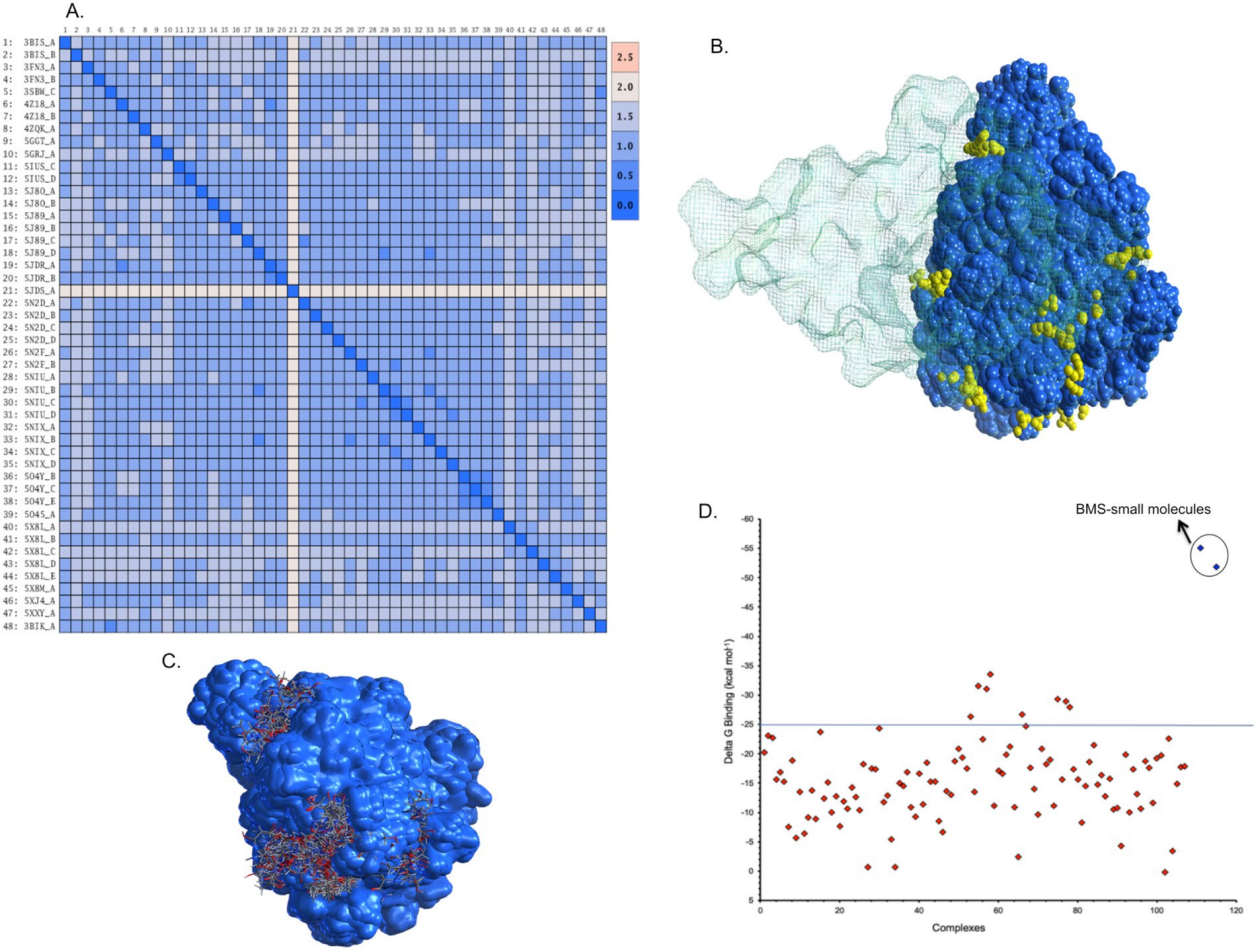
Evaluating the binding of Aurigene-1 against hPD-L1 using computational approaches. (**A**) The root mean square deviation matrix of all the 48 PD-L1 structures in PDB shows that there are no significant conformational differences in PD-L1 while binding to different partners (PD-1, small molecules, monoclonal antibodies and large peptides). (**B**) Identification of different binding pockets (shown as yellow dummy atoms) on the surfaces of all the PD-L1 structures (shown as VdW representations in blue) using Site Finder module in MOE program. Here, the PD-1 protein is shown as a line-surface illustration with ~60% transparency, in order to show the PD-1 binding face on PD-L1. (**C**) The 3D structures showing different docking poses of Aurigene-1 within different binding pockets in PD-L1 (shown as blue surface). (**D**) Comparison of the calculated Delta G binding for all the binding poses of Aurigene-PD-L1 complexes and the top-most pose of BMS-small molecules-PD-L1 complexes (shown with a circle).

We also explored the binding of Aurigene-1 with PD-L1 using computational modeling approaches. For this purpose, we employed all the human PD-L1 chains from the PDB. These include 48 PD-L1 chains from 23 X-ray crystal structures from the PDB (PDB codes: 3BIK, 3BIS, 3FN3, 3SBW, 4Z18, 4ZQK, 5GGT, 5GRJ, 5IUS, 5J8O, 5J89, 5JDR, 5JDS, 5N2D, 5N2F, 5NIU, 5NIX, 5O4Y, 5O45, 5X8L, 5X8M, 5XJ4, 5XXY). This was done so as to account receptor flexibility by exploring all the experimentally determined conformations of human PD-L1 protein. Although these structures include chains from a wide variety of complexes, such as PD-1/PD-L1, mAb/PD-L1, macrocyclic peptide/PD-L1 and small-molecule/PD-L1, their RMSDs were only up to 2.5 Å (Fig. 9A) indicating that these structures were not conformationally very different. We identified all the plausible binding pockets in the 48 PD-L1 structures (Fig. 9B) and docked Aurigene-1 into each one of them using induced-fit method (Fig. 9C), which allows certain levels of binding-site flexibility during docking. Out of the 480 poses of Aurigene-1/PD-L1 complexes from docking, we filtered the poses in which Aurigene-1 was bound at the GFCC’C” face (or PD-1 binding face) of PD-L1 IgV domain and performed a short MD refinement and binding affinity calculations. The binding affinity calculations (Fig. 9D) revealed that only 8 poses had a relative ΔG > 25 kcal mol^−1^. However, the calculated entropy contribution to these poses were >21 kcal mol^−1^. Thus our modeling results, under the defined conditions, also reveal that there is no suitable binding pocket for Aurigene-1 on the PD-L1 interface, which agrees with all the results from our biochemical and cell-based assays that Aurigene-1 does not directly target the PD-1/PD-L1 axis.

### Immunological activity and cytotoxicity of a novel small molecule

**Figure 10:**
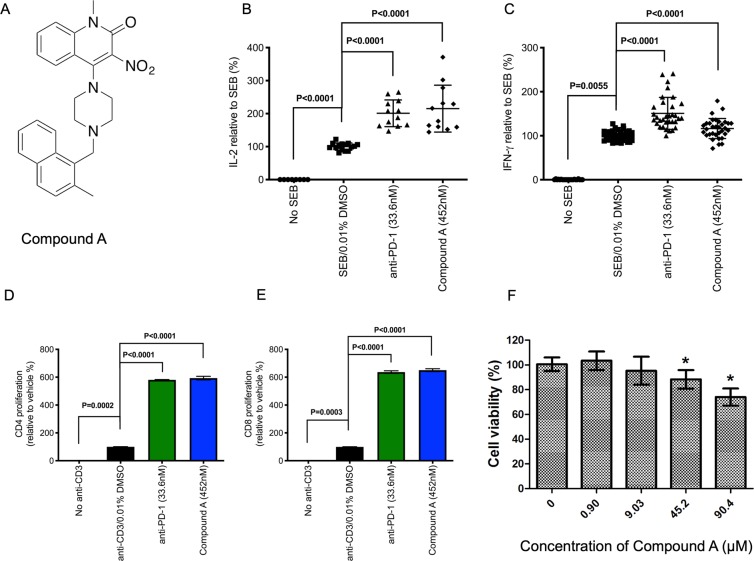
The chemical structure (**A**), immunological activity (**B–E**) and cytotoxicity profile (**F**) of Compound A discovered by our team. Compound A shows immunological activity similar to anti-PD-1. Plots showing production of IL-2 (**B**) and IFN-γ (**C**) by peripheral blood mononuclear cells in response to SEB stimulation and anti-PD-1 or Compound A treatment. (**D**,**E**). Proliferation by CD4+ and CD8+ cells in response to anti-CD3 stimulation and anti-PD-1 or Compound A treatment. Data shows percentage cytokine production or proliferation relative to SEB/anti-CD3. *P < 0.0001 by using one-way ANOVA followed by Dunnett’s multiple comparisons test. Concentration-dependent effects on treating with Compound A on the viability of HepG2 cells are also provided (F). HepG2 cells were treated with various concentrations of Compound A for 24 h, and the cell viability was assessed by the CellTiter-Glo^®^ Luminescent Cell Viability Assay (**A**,**B**) and CCK-8 assay (**C**). Cumulative data from three independent experiments were shown. *ns* = non-significant in comparison with control (DMSO-treated cells). *P < 0.0001 indicates statistical significance calculated by using one-way ANOVA followed by Dunnett’s multiple comparison’s test.

As part of our ongoing research efforts to identify novel small molecule immunomodulators, we recently identified a small molecule, Compound A (Fig. 10A), which was found to exhibit potent immunological activity in our biological assays (U. S. Provisional patent application no. 62/466,243). Compound A induced cytokine production and T cell proliferation (Fig. 10B–E). PBMCs treated with Compound A (at 452 nM) and stimulated with SEB produced similar levels of IL-2 compared to anti-PD-1-treated cells (Fig. 10B). Furthermore, Compound A treatment elicited 2-fold production of IL-2 by peripheral blood mononuclear cells (P < 0.0001, Fig. 10B). We also assessed the ability of Compound A to elicit IFN-γ production by PBMCs (Fig. 10C). IFN-γ production in response to Compound A treatment was approximately 1.2-fold higher than untreated cells (P < 0.0001, Fig. 10C). The induction of IL-2 and IFN-γ production by Compound A-treated cells corresponded with the proliferative ability of similarly-treated T cells, which was similar to anti-PD-1-treated cells (Fig. 10D,E). The viability of HepG2 cells in the presence of different concentrations of Compound A was also evaluated. The cells were treated with Compound A for 24 h and then the cell viability was assessed by the CCK-8 assay. Results from this assay confirmed that treatment of up to a 90 µM concentration of Compound A did not cause any significant effects on the viability of HepG2 cells. Thus, our compound was non-toxic to cells and also immunologically active to induce the production of key cytokines essential for T cell activation. However, our compound did not bind to the PD-1/PD-L1 interface.

## Discussion

Immune checkpoint blockade therapy, a powerful strategy of targeting negative immune regulators to revitalize antitumor activity, has revolutionized the field of cancer immunotherapy. According to Allied Market Research, the current global immune checkpoint inhibitors market is estimated at $10.5 billion and this number is expected to reach ~$57 billion by 2025 (www.alliedmarketresearch.com/immune-check-point-inhibitors-market). Despite remarkable clinical potentials, the mAbs-based therapies face several known limitations associated with neutralizing mAb-based checkpoint therapy^1,30–32^. These limitations could be addressed by a small molecule-based therapy for cancer immunotherapy^54,55^.

In the last 5 years, Aurigene Discovery Technologies Limited^37–40^ and Bristol-Myers Squibb (BMS)^35,41–44^ have separately disclosed several small molecule immunomodulators reportedly targeting the PD-1/PD-L1 axis. However, experimental data for some of these attractive classes of PD-1/PD-L1 inhibitors are not available. The success of small-molecule discovery mainly hinges on understanding the structure-activity-cytotoxicity properties of known molecules that can guide the development of next-generation drug-like inhibitors. Therefore, in this work, we have carried out a comprehensive *in vitro* profiling of the select inhibitors of BMS and Aurigene. For BMS, we chose two small molecule inhibitors, BMS-103 and BMS-142, and a macrocyclic peptide (BMSpep-57). In addition, we selected a promising compound, Aurigene-1, from a recent Aurigene patent that disclosed several peptidomimetic compounds as dual inhibitors of PD-1 and VISTA pathways. Aurigene-1 was reported to rescue 97% of mouse splenocyte proliferation using carboxyfluorescein succinimidyl ester (CFSE) proliferation assay. It should be noted that CA-170, a compound in clinical trial, could be a close derivative of Aurigene-1. We employed a series of biochemical and cell-based bioassays to evaluate the binding, activity and cytotoxicity of the three different classes of promising PD-L1 inhibitors (macrocyclic peptides, peptidomimetic compounds, and non-peptidic small molecules) under similar experimental conditions.

Using three independent binding assays (*i*.*e*. DSF, MST, and SPR), we show that the BMS inhibitors are able to bind specifically to human PD-L1 protein, and not to other related IgV proteins, PD-1 and B7-1. The SPR results herein determined a K_D_ value of 16.10 ± 2.19 nM for BMS-103/PD-L1 complex and 12.64 ± 1.88 nM for BMS-142/PD-L1 complex; whereas our MST data revealed a binding affinity of 44 ± 13 nM and 13.2 ± 1.5 nM for BMS-103 and BMS-142, respectively, for binding with PD-L1. These BMS inhibitors also exhibited concentration-dependent inhibitory activity in the PD-1/PD-L1 biochemical ELISA, with an IC_50_ value of 79.1 nM (for BMS-103) and 96.7 nM (for BMS-142). We have compared the ability of the BMS compounds (BMS-103, BMS-142 and BMSpep-57) to facilitate T cell function mirrored by IL-2 production. Although production of IL-2 is not the only indicator of T cell function, loss of this phenotype is a significant hallmark of T cell exhaustion^56^. In this regard, we used this read-out to evaluate the efficacy of these compounds. Furthermore, and importantly, we investigated whether the induction of IL-2 production by these compounds correlated with interference with PD-1 signaling.

Results obtained from measuring the concentration of IL-2 in cell culture supernatants showed that α-PD-1 or α-PD-L1 neutralizing mAbs induced increased IL-2 production by stimulated PBMCs. This was expected due to the success of checkpoint inhibitor mAbs such as anti-CTLA-4 and anti-PD-1 alone or in combination in restoring T cell responses and control of tumor growth^6^. Compared to the neutralizing antibodies we found that BMS compounds such as BMS-103 and BMSpep-57 triggered the production of modest levels of IL-2 at certain concentrations. Interestingly we found that the ability to induce IL-2 production in T cells does not always correspond with the interference of these compounds with the PD-1/PD-L1 interaction (Fig. 4). The NFAT activity (luminescence) of T cell receptor (TCR)-activated PD-1-expressing, similarly treated Jurkat cells was used to evaluate PD-1/PD-L1 binding. We found that high concentrations of BMS-142 (24 µM and 48 µM) that are possibly unsuitable for long incubation periods required for SEB stimulation sometimes interacted weakly with PD-1/PD-L1, although this was not statistically significant (Fig. 5A). This occurrence is not surprising as small molecule interference with PD-1-PD-L1 interaction is likely to be dependent on concentration and occupancy of the appropriate binding sites. However, this was not the case with BMS-103 as both low and high concentrations used were insufficient to promote NFAT activity of treated Jurkat cells (Fig. 5A). These observations indicate that the structural differences in the two BMS small molecules may play a more direct and integral role in interference with PD-1/PD-L1 interaction compared to concentration. The main challenge with BMS-103 and BMS-142 stems from their acute cytotoxic nature, as confirmed by our cell-viability experiments (Fig. 7). However, recently, BMS disclosed two optimized small-molecule inhibitors (BMS-1001 and BMS-1166) from the same structural scaffold as BMS-103 and BMS-142 that displayed low toxicity and enhanced cell-based activity^40^. Dimer inducing small molecules, such as the BMS-103 and BMS-142, are new to the immune checkpoints research area. Very recently, Perry *et al*.^57^ reported a number of novel chemotypes of small molecule fragments that also induced dimerization of two free PD-L1 proteins and prevented the PD-1/PD-L1 interactions. Therefore, it is possible that ‘dimer-locking’ is one of the best viable options for targeting the high-affinity and high-avidity involved immune checkpoint protein-protein interactions. Gaining more in depth understanding of molecular mechanism behind this complicated molecular sandwich (i.e., PD-L1-Small-molecule-PD-L1) could facilitate the rational design of small molecules for specifically inducing ‘dimer-locking’ in immune checkpoints rather than accidental discovery.

In the case of Aurigene-1, our experiments consistently demonstrate that it did not bind to PD-L1 (or PD-1) surface or show any activity in biochemical ELISA (Figs 2 and 3). Our cell-based experiments confirm that Aurigene-1 neither induced IL-2 cytokine release nor showed any NFAT activity of Jurkat cells, at the analyzed concentrations, when compared to the background levels (Fig. 6). Our molecular computational models also suggest there is no suitable site at the interface of PD-L1 for Aurigene-1 to bind (Fig. 9). Thus, our work demonstrate that Aurigene-1 does not directly bind or interfere with the PD-1/PD-L1 axis; and it is possible that it acts through a different mechanism/target that inhibits PD-1 signaling.

It is not surprising that a small-molecule could indirectly target the PD-1 signaling, which involves a complex stream of downstream processes. As described above, our small molecule, Compound A, was also identified as a potent immunomodulator that is able to induce cytokine production (IL-2 and IFN-γ) and T cell proliferation, at levels comparable to pembrolizumab – an approved anti-PD-1 human mAb for treating metastatic melanoma (Fig. 10). The compound was also found to have minimal effect on the viability of HepG2 cells, which satisfied an essential preclinical requirement for a small molecule. However, similar to Aurigene-1, the mode-of-action of our compound is also not clear, as it did not bind to either of the PD proteins. Nevertheless, its strong immunological activity makes it an ideal compound for lead optimization.

## Materials and Methods

### Purification of recombinant human PD-L1

The gene of extracellular domain of mature form of human PD-L1 (residues 18–134) was cloned into pET21b vector (Novagen, US). The plasmid bearing the gene of interest was transformed into chemically competent BL21 (DE3) cells. The protein expression was carried out for 4 hours at 30 °C after inducing the protein with 1 mM IPTG. The purification strategy was adapted from Cheng *et al*.^58^. The inclusion bodies were harvested and the protein was unfolded with 50 mM Tris-HCl, 200 mM NaCl, 2 mM EDTA, 10 mM β-mercaptoethanol and 6 M Gu-HCl buffer. The refolding was performed by drop-wise dilution in 50 mM HEPES, pH 7.5, 500 mM L-arginine, 1 mM glutathione, 1 mM glutathione disulfide, 100 mM NaCl, followed by dialysis against 50 mM HEPES, 50 mM NaCl, 1 mM TCEP. The purification was completed by size exclusion chromatography (SEC) on Superdex75 (10/30) column (GE Healthcare, Canada) to ensure homogeneity. The purity and refolding was assessed by SDS-PAGE and NMR (data not shown).

### Differential scanning fluorimetry (DSF)

For DSF assay,‘ PD-L1 was used in a final concentration of 10 µM, and the tested compounds dissolved in DMSO were added to the protein sample to the final concentration of 50 µM^59^. The protein incubated with DMSO alone was included as a control. The concentration of DMSO or compound in DMSO did not exceed 5% of protein sample. After an incubation period (5 min at room temperature) for protein equilibration with compound, SyproOrange dye (Thermo Fisher Scientific, USA) was mixed with the protein. Prior to use, the dye stock was diluted 1:50 (100x) and used immediately while protecting from light to reduce photobleaching. The optimal dilution of the dye in the assay was determined empirically with a 5x dilution for the final assay. The thermal denaturation assay was performed in a total volume of 40 µl. All samples were run in triplicates. The thermal scan was conducted from 25 to 95 °C, at 0.5 °C/min (Mastercycler Gradient 96, Eppendorf, USA). The melting point (T_m_) was calculated by fitting the raw fluorescence data over the temperature using the Boltzmann equation in GraphPad Prism program (GraphPad Prism 7 for Windows, GraphPad Software, USA).

### Assessment of compounds binding to PD-L1 using microscale thermophoresis

The PD-L1 protein was labelled using Monolith His-Tag Labeling Kit RED-tris-NTA (NanoTemper Technologies, Germany) following the standard protocol. The measurements were performed on a NanoTemper Monolith NT.115 instrument (NanoTemper Technologies, Germany). His-labeled PD-L1 was used in a concentration of 60 nM for all binding experiments. The compounds were dissolved in DMSO. Serial dilutions were made in DMSO and then 1 µl of each dilution was mixed with 19 µL of labelled protein in 50 mM HEPES, pH 7.5, 50 mM NaCl buffer, aiming for the final DMSO concentration of 5% in each sample. The samples were loaded into Monolith NTTM Standard Treated Capillaries (NanoTemper Technologies, Germany). In order to find the best thermophoretic setting, the binding of compounds at different infrared laser powers were analyzed, choosing the heating power with the best signal to noise ratio. All measurements were performed at 40% LED power, 30 sec laser-on time and 5 sec of final laser-off time for back diffusion.

## SPR analysis

The kinetic analysis for the interaction of small molecules (BMSpep-57, BMS-103, BMS-142 and Aurigene-1) with PD-L1 or B7.1 was performed on a Biacore T200 (GE Healthcare Bio-sciences, Sweden) using Series S Sensor Chip Protein A (GE Healthcare Bio-sciences, Sweden, catalog # 29-1275-56). Briefly, Fc-PD-L1 (PD1-H5258, Acro Biosystems, MA, USA) or Fc-B7.1 (B71-H5259, Acro Biosystems, MA, USA) was diluted using 1x PBS-P+ buffer (phosphate-buffered saline 1×, Hyclone, Catalog number SH30256.01, enriched with 0.05% polysorbate-20) containing 2% DMSO (Sigma-Aldrich, ON, Canada) and captured as the ligand (i.e. the target of small molecules) on protein A chip surface following Biacore T200 build-in protocol for kinetic/affinity analysis at a concentration of 5 μg/ml or 4 μg/ml, respectively. The contact time and flow rates were set as 60 sec and 10 μl/min, respectively, for the ligand capture. Each experiment was performed using a pair of flow cells, one having ligand captured and the other keeping as reference. BMSpep-57, BMS-103 and BMS-142 were made into a stock solution of 10 μM using DMSO, while Aurigene-1 was made into stock solution of 10 mM using DMSO. Each of the compounds were diluted 50x using 1x PBS-P+ buffer and then made serial dilution using 1x -PBS-P+ buffer with 2% DMSO to get test concentrations of BMS compounds as 0, 12.5, 50 and 200 nM, and that of Aurigene-1 as 0, 12.5, 50 and 200 μM. His-CTLA4 (CT4-H5229, Acro Biosystems, MA, USA) protein was diluted using 1x PBS-P+ with 2% DMSO buffer to get final concentrations as 8.12, 32.47, and 129.8 nM. Analytes (BMSpep-57, BMS-103, BMS-142, Aurigene-1, His-CTLA4 and His-PD1) were allowed to flow through both ligand-captured flow cells and reference flow cells at the same rate (30 μl/min) and contact time (120 sec). Solvent correction was included to avoid the impact of DMSO on surface plasmon effect during binding analysis. Extra wash of the flow system using 50% DMSO in 1x PBS-P+ buffer was allowed to control carry over effects. For regeneration of surface after each cycle (one-time ligand capture and binding of analyte at a single concentration) was carried out using 10 mM glycine pH 1.5 (GE Healthcare Bio-sciences, Sweden) at a flow rate of 30 μl/min for 30 sec. Kinetic analysis was performed at 25 °C. Maximum binding response (R_max_) and equilibrium dissociation rate constant (K_D_) values were calculated using Biacore T200 Evaluation software following the 1:1 Langmuir binding model with global fit parameters for solvent-corrected sensograms.

### NMR spectroscopy

NMR samples contained 12 µM of protein, 200 µM of compound in 1x PBS (phosphate-buffered saline buffer, pH 7.5) and 0.25 mM 2,2-dimethyl-2-silapentane-5-sulfonate-d6 sodium salt (DSS-d6) as an NMR internal reference in 95/5% H2O/D2O or 100% D2O. Stock solution of 50 mM of compound was prepared in DMSO-d6 (Cambridge Isotopes Inc.) and added in aliquots to the protein sample, thus the concentration of DMSO-d6 did not exceed 5% of the sample volume. NMR spectroscopy was performed at the Nanuc NMR Centre on a 2.2 K pumped-800 MHz Oxford magnet updated to a Bruker Neo-Advance IV console equipped with a 5 mm cryoprobe and SampleJet robotics.

### PD-1/PD-L1 ELISA competition assays

High-binding 96 well plates (Corning Inc, Kennebunk, ME) were coated with 0.1 µg/100 µL/well of Fc-tagged human PD-L1 (R&D Systems, Minneapolis, MN). Standard curves were generated in each plate with 0–1,000 ng/mL biotinylated human PD-1 (Acro Biosystems). Test compounds or peptides were diluted in buffer (PBS, 0.5% BSA, 0.05% Tween 20, 0.5% DMSO) then pre-incubated in the coated wells for 30 minutes before adding 150 ng/mL biotinylated human PD-1. These ligand concentration used for screening was chosen based on the linear range of the standard curve (concentration which fall into >50% of linear range in the standard curve). After one hour incubation with the ligand, the plate was washed,and the bound ligand was detected with the addition of streptavidin-HRP (R&D Systems) followed by development with TMB solution (SeraCare Life Sciences Inc, Milford, MA). Binding was quantified in relation to the ΔA_450_ (A_450_ from sample - A_450_ from uncoated background) of the standard curve in each assay plate using a four parameters logistic nonlinear regression (GraphPad Prism v 7.03). Aurigene-1 compound was also tested in a reverse configuration of PD-1/PD-L1 ELISA assay, where 0.1 µg/100 µL/well of Fc-tagged human PD-1 (Acro Biosystems) was coated on the plates and the biotinylated human PD-L1 (Acro Biosystems) was used to study binding. Further details of this assay are provided in Supplementary Text, S.Text 1.

### Blood samples and cell isolation

PBMC samples from HIV, HCV and HBV seronegative individuals were used for this study. The appropriate Institutional Review Boards at the University of Alberta approved the studies (Pro00063463 and Pro00046064). All study participants gave written informed consent to participate in this study. All methods were performed in accordance with the relevant guidelines and regulations. Whole blood was processed by density gradient centrifugation using Ficoll-Paque PREMIUM (GE Healthcare). Peripheral blood mononuclear cells (PBMCs) were collected and washed twice with pre-warmed RPMI-1640 medium (Sigma-Aldrich) containing 10% fetal bovine serum (FBS), L-glutamine (300 mg/L) and antibiotics (1% Penicillin/Streptomycin). The cells were cryopreserved in FBS containing 10% DMSO (Sigma-Aldrich) in liquid nitrogen until use. In some cases, fresh PBMCs were used for experiments.

### Antibodies and small molecules

Human mAbs that target the checkpoint inhibitors Programmed Cell Death-1 (PD-1) and its ligand, PD-L1 were used in this study. Clinical grade anti-PD-1 (pembrolizumab, (Keytruda)) and research grade anti-PD-L1 mAb (LEAF™ Purified anti-human CD274, clone 29E.2AE, Biolegend), were used in this study. The following compounds were used in this study: BMS-103, BMS-142, BMSpep-57 and Aurigene-1. Aurigene-1 was purchased from a supplier and obtained in 95% purity. Identity of the received Aurigene-1 was confirmed by NMR spectroscopy (Supplementary Figs S10 and S11) and Mass-spectroscopy (Supplementary Fig. S12). The BMS small molecules, BMS-103 and BMS-142 were purchased from TC Scientific (Edmonton) and obtained in 94% and >99% purity, respectively. BMSpep-57 (with 95.05% purity) was purchased from Selleck Chemicals LLC. Compound #14 (with purity >95%), a known sulphonamide-based inhibitor of the cccDNA of Hepatitis B virus^60^, was used as a negative control in this study. We also tested the activity of one our screening products, Compound A, using cytokine ELISAs (IL-2, IFN-γ) and T cell proliferation assays. The structural characterization of compound A was performed using Liquid chromatography-mass spectroscopy (LCMS) analysis and NMR technique (data provided in Supplementary Figures, Figs S13–S16). Stock solutions of all small molecule inhibitors were prepared in DMSO based on solubility requirements. Stock solutions were further diluted in assay media to obtain working concentrations (ranging from approximately 517 nM to 60 μM). Similar amounts of DMSO were added to mAb-treated positive control and untreated control wells.

### Stimulation of peripheral blood mononuclear cells

2.5 × 10^5^ PBMCs per well were plated in a 96 U-bottomed plate in triplicate and stimulated with 100 ng/ml Staphylococcal Enterotoxin B (SEB) (Sigma) for 72 h at 37 °C, 5% CO_2_ in the presence of small molecule inhibitors (up to 48 μM), human α-PD-1 mAb (33.6 or 66.1 nM) or human α-PD-L1 mAb (90.9 or 181.8 nM, Biolegend). Untreated or unstimulated PBMCs were used as positive and negative controls respectively. In order to confirm PD-1 and PD-L1 expression in response to peripheral blood mononuclear cell stimulation, the expression of PD-1 and PD-L1 by CD4+ and CD8+ T cells and PD-L1 by CD11b+ and CD11c+ APCs was analysed by flow cytometry (Supplementary Fig. S9). For T cell proliferation assays, peripheral blood mononuclear cells were labelled with a 1.25 μM CFSE concentration (ThermoFisher Scientific) before stimulation with a-CD3/CD28 in the presence of small molecule inhibitors for 4 days.

### IL-2 and IFN- γ ELISA

Cell culture supernatants from SEB-stimulated PBMCs were harvested and IL-2 or IFN-γ production was measured using ELISA (Human DuoSet ELISA kits, R&D Systems) according to manufacturer’s instructions.

### Bioluminescent cell-based assay for PD-1/PD-L1 inhibitor screening

PD-1/NFAT reporter Jurkat cells and recombinant Chinese Hamster Ovary (CHO-K1) cells that constitutively express PD-L1 and an engineered TCR activator were purchased from BPS Bioscience (CA, USA). To screen small molecule inhibitors, Jurkat cells were co-cultured with CHO cells according to manufacturer’s instructions. Briefly, 3 × 10^4^ Jurkat cells were pre-incubated with varying concentrations (up to 48 μM) of small molecule inhibitors or monoclonal antibodies (anti-PD-1 (33.6 or 66.1 nM) or PD-L1; (90.9 or 181.8 nM) at a 1:1 volume for 30 min at 37 °C followed by co-culture with 5 × 10^4^ CHO cells per well for 6 h at 37 °C. After incubation the ONE-Step luciferase assay (BPS Bioscience, CA, USA) was performed by adding the luciferase reagent to treated wells and untreated controls for 30 min followed by luminescence measurement. The fold induction of NFAT luciferase reporter expression is calculated as background-subtracted luminescence of treated well/average background-subtracted luminescence of untreated control wells. For counter screening experiments PD-1 negative Jurkat T cells were used.

### Evaluation of acute cytotoxicity of small molecules in Jurkat and CHO cells

The acute toxicity (viability by ATP) of the small molecules was tested *per se* in both the Jurkat and CHO cells that were used in bioluminescent cell-based assay for PD-1/PD-L1 inhibitor screening. Test compounds were prepared in DMSO and subsequently diluted in culture medium with a 1% final DMSO concentration. Briefly, Jurkat cells were seeded in solid white 96-well microplates at a density of 20,000 cells per well in 100 µl of medium, treated with the test compounds and incubated for 24 h. In the case of CHO cells, the cells were plated in solid white 96-well microplates at a density of 20,000 per well and allowed to adhere for 24 h. The cells were washed once, treated with test compounds and incubated for 24 h. Following the incubation period, the CellTiter-Glo^®^ Luminescent Cell Viability Assay (#G7570, Promega, Madison, WI) was performed following the manufacturer’s protocol. Luminescence values were presented as a percentage of the vehicle control and expressed as mean ± SD. All treatments were carried out in triplicates, and the assay was repeated three independent times. Statistical analysis was carried out using GraphPad Prism 6 software.

### Evaluation of acute cytotoxicity of small molecules in human hepatoma (HepG2) cells

The acute cytotoxicity of the test compounds was also evaluated by administering the test compounds to human hepatoma (HepG2) cells and assessing their viability (metabolic viability) using the CCK-8 assay (based on WST-8). HepG2 cells are commonly used in the early screening stages for new chemical entities, and the CCK-8 assay has been well established as a sensitive and reliable indicator for cellular metabolic activity (viability) for this purpose. HepG2 cells were obtained from ATCC (HB-8065; Manassas, VA) and maintained in DMEM culture medium in a water-jacketed incubator (5% CO_2_, 37 °C). Test compounds were prepared in DMSO and subsequently diluted in culture medium with a 1% final DMSO concentration. Cells were plated in 96-well plates at a density of 10,000 per well and allowed to adhere for 24 h. The cells were washed, and treated with test compounds and incubated for 24 h. At this time point, cells were treated with WST-8 based on the manufacturer’s protocol (CCK-8 kit, Dojindo Molecular Technologies, Rockville, MD). Cell viability calculations were also carried out according to the manufacturer’s protocol, which included accounting for potential interference from test compounds, and comparisons to appropriate controls. GraphPad Prism was used for data analysis and graphical representation.

### Statistical analysis

Statistical analysis was performed using GraphPad Prism v7.03 or above (GraphPad Software Inc., La Jolla, CA) and described details in figure legends and/or experimental details section. Data shown are mean ± SD. A p value < 0.05 was considered significant.

### Computational details

All docking calculations in this study were carried out using Molecular Operating Environment, MOE 2018.01, package^61^. The three dimensional structures of the BMS-103/PD-L1 and the BMS-142/PD-L1 complexes were modelled using the pharmacophore fingerprints of BMS-202, belonging to the same (2-methyl-3-biphenylyl)methanol scaffold as BMS-103 and BMS-142, and whose binding mode with human PD-L1 has been experimentally resolved (PDB: 5J89)^47^. Pharmacophore placement method (for initial docking) and induced-fit method (for secondary refinement) in MOE were used for docking of BMS small molecules in this study. Since there is no information about the binding site and/or binding mode of Aurigene-1 with human PD-L1 protein, we explored the plausible binding pockets in all the human PD-L1 chains available in the PDB (~48 structures), as described in the Supplementary Text, S.Text 2. Two stage molecular docking calculations, initial induced-fit docking and followed by refinement based on AffinitydG scoring function, was carried out using MOE that resulted in a total of 480 Aurigene-1/PD-L1 complexes. Among these poses, only those in which Aurigene-1 bound at the GFCC’C” face (or PD-1 binding face) of PD-L1 IgV domain were selected for further MD refinement and analyses.

The structures of all the small-molecule/PD-L1 complexes (such as BMS-103/PD-L1, BMS-142/PD-L1, and Aurigene-1/PD-L1) obtained from preliminary docking calculations were solvated in a 12 Å wide periodic cubic box of explicit TIP3P water molecules and subjected to 2 ns long MD simulations using the Amber 14 package and the Amberff14SB force fields^62^, as described in Supplementary Text, S.Text 2. For each MD trajectory produced, 10 snapshots collected at a regular interval of 100 ps from the last 1 ns time scale were employed for calculating the relative binding affinities using the MM-GBSA approach. For only the select systems, with relative affinity >25 kcal mol^−1^, the entropy contribution was calculated using normal mode analyses. For all the MM-GBSA calculations in this work, we employed the implicit GB solvent model, GB-Neck2 (igb = 8)^63^ for calculating the electrostatic solvation-free energy and the salt molar concentration was set to 0.15 M. All MMGBSA analyses in this study were carried out using the MMPBSA.py script included in the AmberTools. More details about the computational calculations are provided in Supplementary Text, S.Text 2.

## Supplementary information

Supplementary file

## Data Availability

The data that support the finding of this study are available from the corresponding author upon request.
