# Supplementary material for: Comprehensive in vitro characterization of PD-L1 small molecule inhibitors
Source: Sci Rep. 2019 Aug 27;9:12392. doi: 10.1038/s41598-019-48826-6 (PMC6712002; doi:10.1038/s41598-019-48826-6)
Supplement: Supplementary file 1 — Supplementary file [file 41598_2019_48826_MOESM1_ESM.docx]

**Comprehensive *in vitro* characterization of PD-L1 small molecule inhibitors**

**Supplementary Information**

Aravindhan Ganesan^1^, Marawan Ahmed^1^, Isobel Okoye^2^, Elena Arutyunova^3^, Dinesh Babu^1^, William L. Turnbull^4^, Joydeb Kumar Kundu^5^, Justin Shields^6^, Katharine Cheryl Agopsowicz^7^, Lai Xu^2^, Yasser Tabana^1^, Nutan Srivastava^1^, Guangzhi Zhang^2^, Tae Chul Moon^1^, Alexandr Belovodskiy^5^, Mostofa Hena^5^, Appan Srinivas Kandadai^5^, Seyedeh Nargess Hosseini^4^, Mary Hitt^6,7,8^, John Walker^8^, Michael Smylie^8^, Frederick G.West^4,7^, Arno G. Siraki^1,7^, M. Joanne Lemieux^3,7^, Shokrollah Elahi^2,6,7,9^, James A. Nieman^5^, D. Lorne Tyrrell^5,6,9^, Michael Houghton^5,6,9^ and Khaled Barakat^1,5,6,7*^.

^1^Faculty of Pharmacy and Pharmaceutical Sciences, University of Alberta, Edmonton, AB, Canada.

^2^Department of Dentistry, Faculty of Medicine & Dentistry, University of Alberta, Edmonton, AB, Canada.

^3^Department of Biochemistry, Faculty of Medicine & Dentistry, University of Alberta, Edmonton, AB, Canada.

^4^Department of Chemistry, Faculty of Science, University of Alberta, Edmonton, AB, Canada.

^5^Li Ka Shing Applied Virology Institute, University of Alberta, Edmonton, AB, Canada.

^6^Li Ka Shing Institute of Virology, University of Alberta, Edmonton, Alberta, Canada.

^7^Cancer Research Institute of Northern Alberta, University of Alberta, Edmonton, Alberta, Canada.

^8^Department of Oncology, Faculty of Medicine & Dentistry, University of Alberta, Edmonton, AB, Canada

^9^Department of Medical Microbiology and Immunology, Faculty of Medicine & Dentistry, University of Alberta, Edmonton, AB, Canada.

*Corresponding author

[kbarakat@ualberta.ca](mailto:kbarakat@ualberta.ca)

**Fig. S1:** **DSF of PD-1 in the presence of Aurigene-1 (A) and the binding affinity of PD-L1 to compound #14 as determined by MST (B)**. (A) The presence of Aurigene-1 did not induce thermal stability to the human PD-1 protein, which suggests there is no binding event between Aurigen-1 and PD-1 protein. (B) As expected, compound #14 shows no binding to PD-L1 in the MST experiment. The difference in normalized fluorescence F_norm_ [‰] was plotted against MST T-Jump.


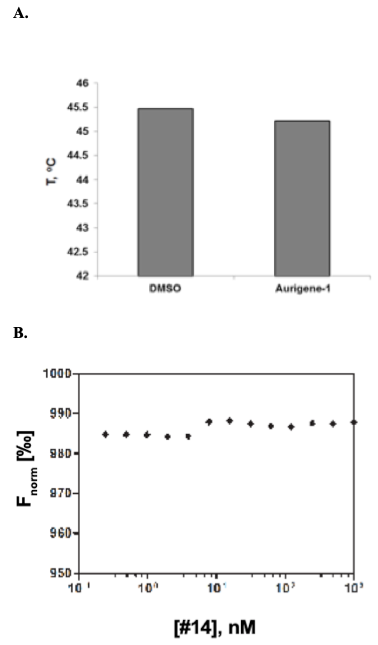


**Fig. S2:** **Binding of the selected molecules with Fc-PD-L1 or Fc-B7-1 measured using SPR technique.** **(a)** His-PD-1~~,~~ was allowed to flow over Fc-PD-L1 captured on Series S Protein A chip as well as on a blank reference cell on Biacore T200. **(b)** BMSpep-57, BMS-103, BMS-142, and His-CTLA4 were allowed to flow over Fc-B7.1 captured on a flow cell as well as on a reference cell of Series S Sensor Chip Protein A at indicated concentrations, and the R_max­_ and K_D_ values were determined by Biacore Evaluation software. The solvent-corrected sensograms were presented as representative one from two separate assays showing similar results.


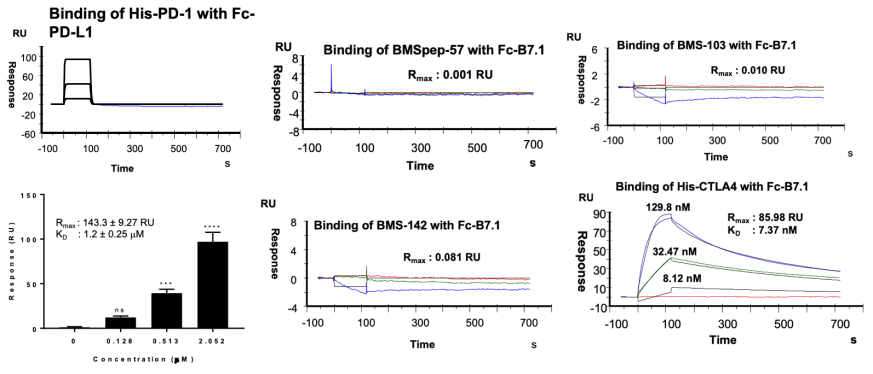


**Fig. S3: STD and WaterLOGSY experiments of PD-L1 binding to Aurigene-1**. Blue - 1D spectrum of Aurigene-1, Red and Green -STD and WaterLOGSY spectra respectively of 12 μM of PD-L1 with 200 μM of Aurigene-1


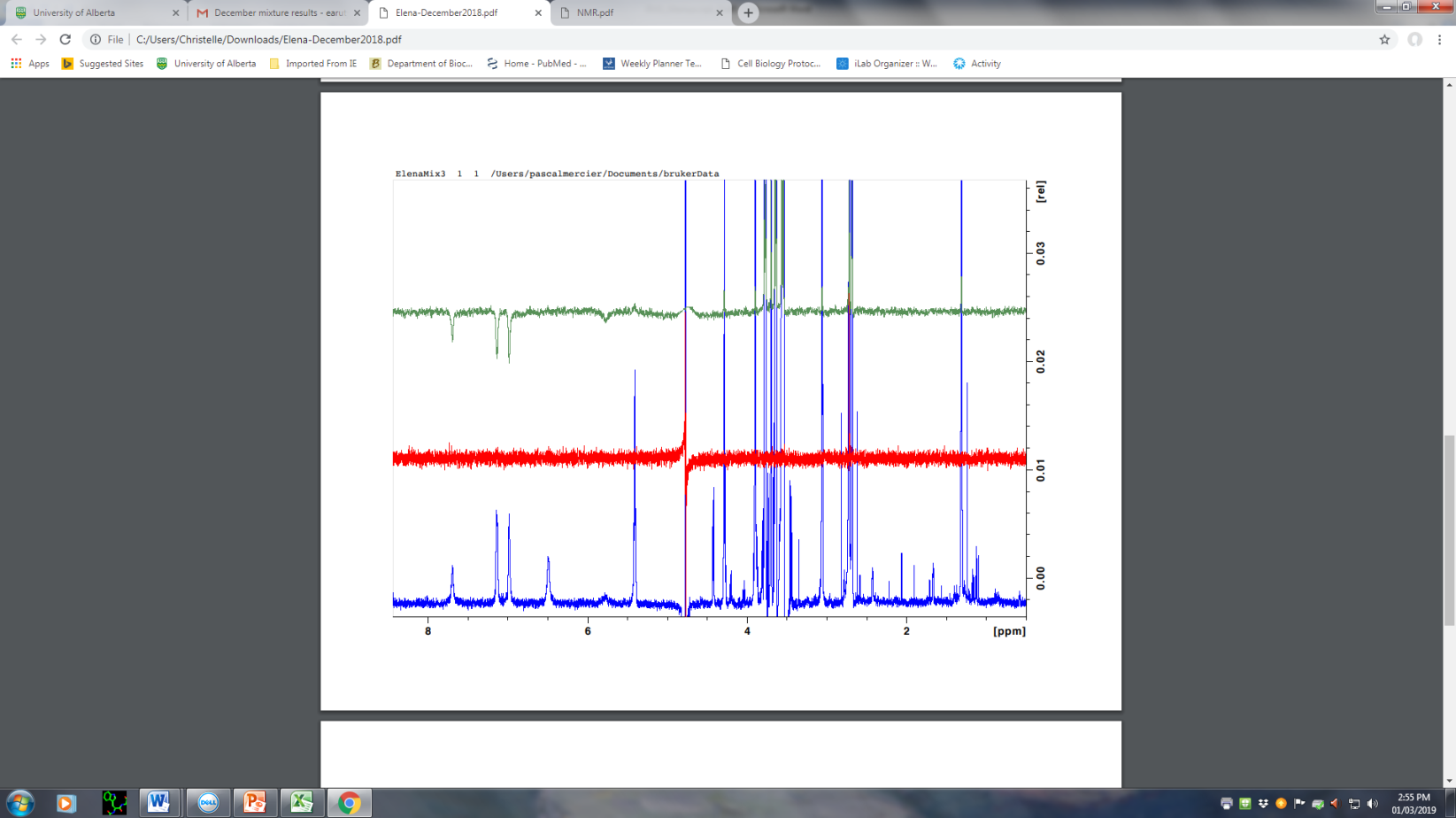


**Fig. S4: Inhibition of binding by αPD-1 antibody in ELISA. Antibody was pre-incubated in plates coated with either PD-L1 or PD-1, followed by incubation with the corresponding biotinylated ligand (PD-1 or PD-L1)**. The PD-1:PD-L1 binding inhibition percentage was calculated from the difference in the amount of biotinylated ligand bound in the presence or absence (vehicle alone) of the antibody. In the absence of the antibody, binding inhibition was normalized to 0%. Where signal was reduced below the level of the standard curve, inhibition was set to 100% (as is the case for 4 mg/ml antibody in PD-1 coated plates). Data represent mean ± SD from two independent experiments, except 0.1 mg/ml antibody where n=1. Each independent experiment was run duplicates in the presence of antibody, or 8 technical replicates for 0 mg/ml controls.

**
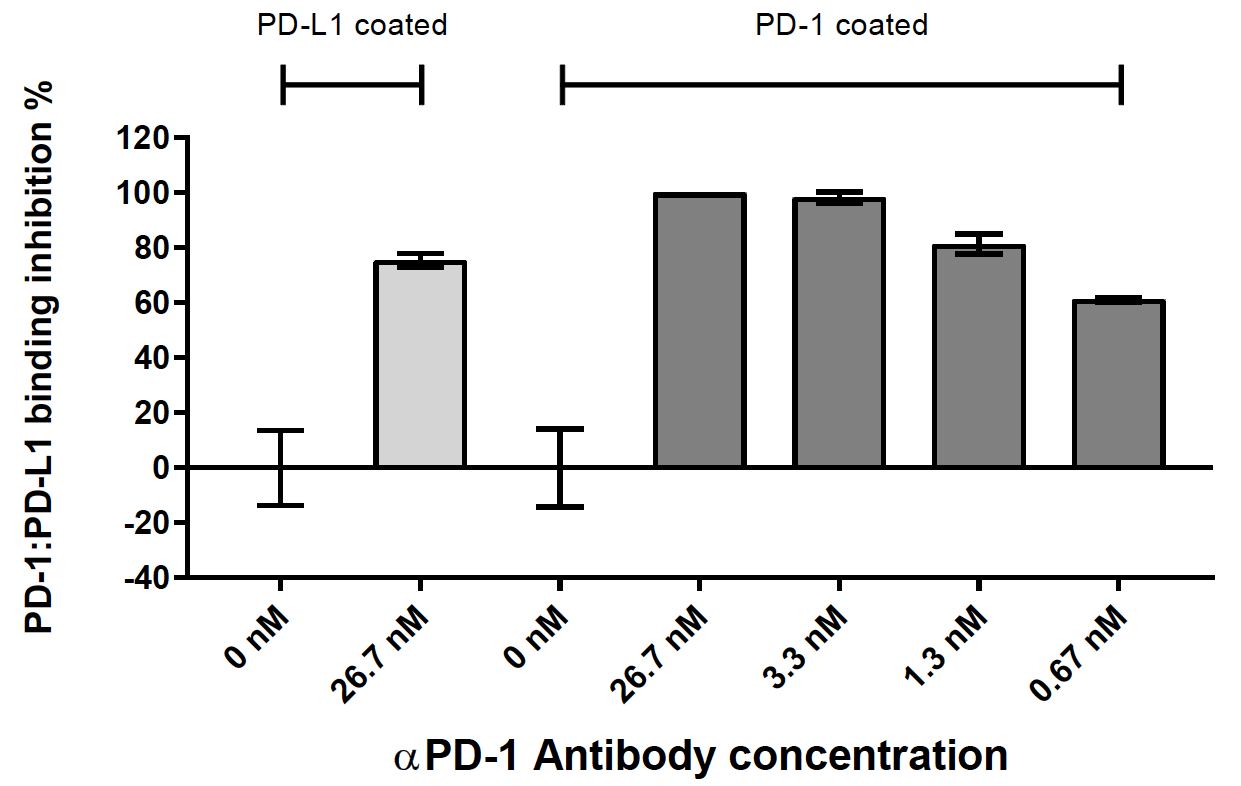
**

**Fig. S5: Inability of Aurigene-1 to inhibit PD-1:PD-L1 binding in a reverse configuration ELISA .** Aurigene-1 was pre-incubated in plates coated with PD-1 followed by incubation with biotinylated PD-L1. The PD-1:PD-L1 binding inhibition percentage was calculated from the difference in the amount of biotinylated PD-L1 bound in the presence or absence (vehicle alone) of the compound. In the absence of the compound, binding inhibition was normalized to 0%. Data represent mean ± SD from three independent experiments, each independent experiment was run triplicates.


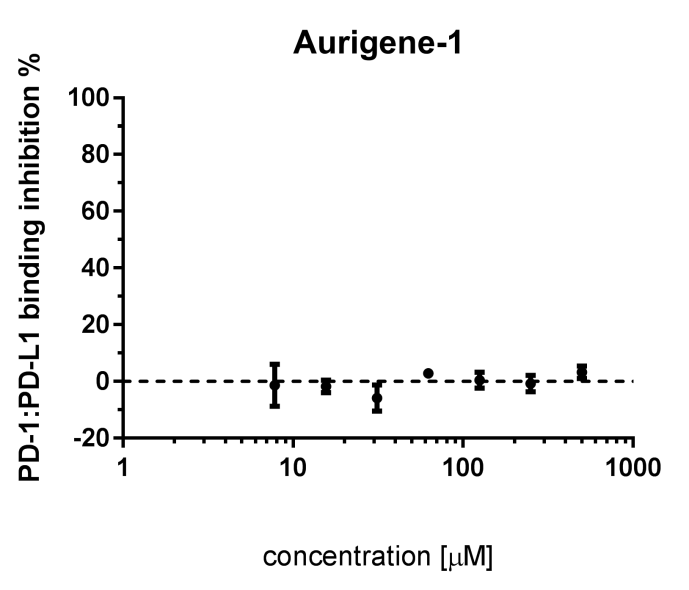


**Fig. S6: Counter-screening assay results showing NFAT-luciferase reporter activity elicited by PD-1 negative NFAT Reporter Jurkat cells treated with neutralising antibodies (PD-1 or PD-L1) or BMS compounds and co-cultured with TCR activator/PD-L1-CHO cells**. Fold luminescence of Jurkat cells treated with antibodies (a-PD-1 (66.1 nM), a-PD-L1 (181.8 nM) or two-fold concentrations of the indicated BMS compounds and negative control compound #14 compared to the untreated (cells only). P-values indicate statistical significance calculated using the Kruskal-Wallis test followed by Dunn’s multiple comparisons test. Cumulative data showing mean ± SD from three experiments shown.

**
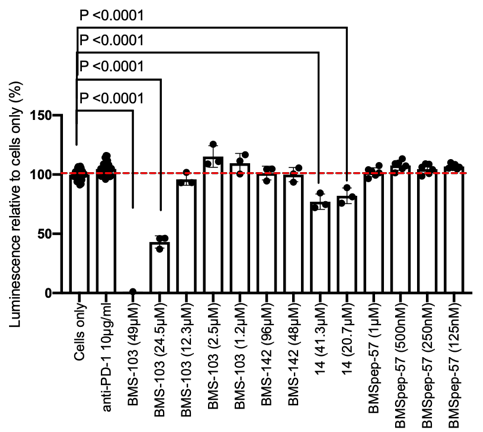
**

**Fig. S7: Counter-screening assay results showing NFAT-luciferase reporter activity elicited by PD-1 negative NFAT Reporter Jurkat cells treated with neutralizing antibodies (PD-1 or PD-L1) or Aurigene-1 and co-cultured with TCR activator/PD-L1-CHO cells**. Fold luminescence of Jurkat cells treated with antibodies (a-PD-1 (66.1 nM), a-PD-L1 (181.8 nM) or two-fold concentrations of the indicated Aurigene compounds compared to the untreated (cells only). Cumulative data showing mean ± SD from three experiments shown.

**
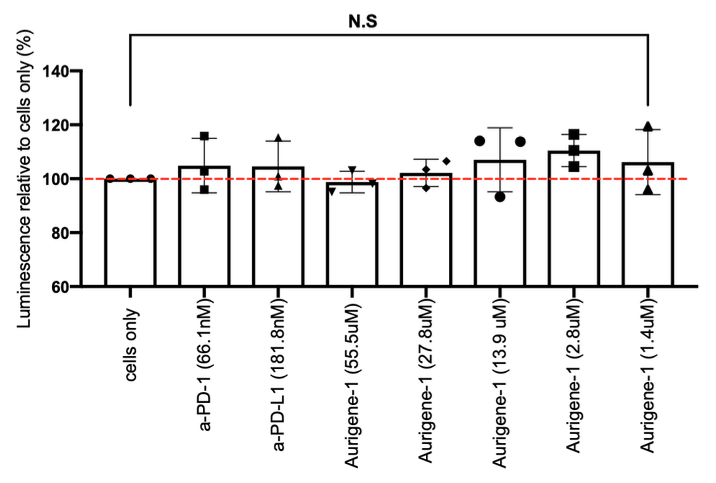
**

**Fig. S8: The binding of macrocyclic BMSpep-57 with PD-L1 surface (PDB 5O4Y).** The BMSpep-57 inhibitor (shown as ball and stick representation in red) binds at the PD-1 binding surface of PD-L1 (shown as a cartoon representation in blue) thereby inhibiting the PD-1/PD-L1 axis. The PD-1 protein, in the binding pose with PD-L1, is shown as a wired surface with 75% transparency.

**
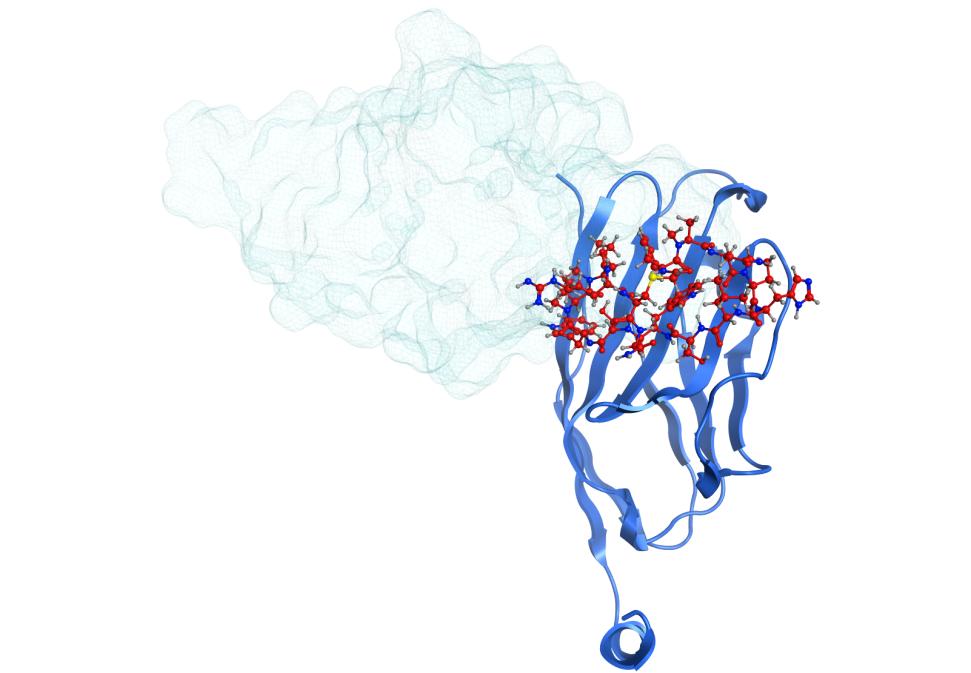
**

**Fig. S9:** Representative flow cytometry plots showing the expression of PD-L1 by CD11b+ and CD11c+ APCs (A) and PD-1 and PD-L1 by CD4+ and CD8+ T cells (B). PD-1/PD-L1 expression is based on gated live lymphocyte/APC populations.

**
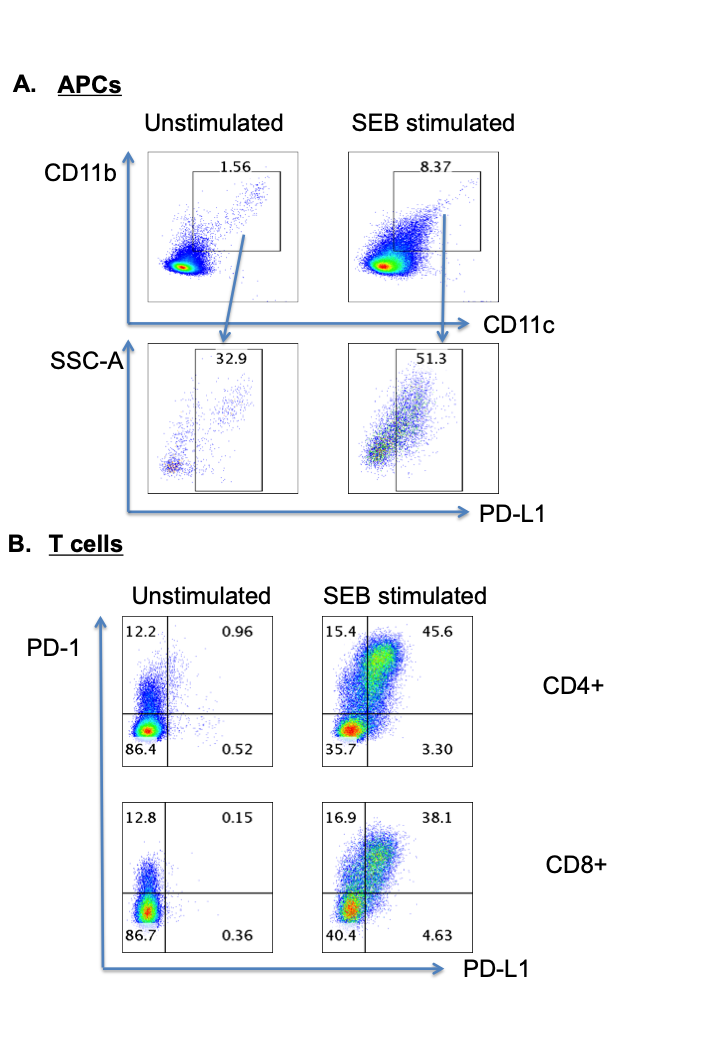
**

**Fig. S10: Comparison of ^1^H NMR spectra of Aurigene-1 provided by the supplier (top) and that recorded by the authors (bottom).** The ^1^H NMR spectrum of Aurigene-1 was recorded on a Varian Inova 500 MHz spectrometer in DMSO-*d_6_* and referenced to the residual solvent signal. ^1^H NMR (500 MHz, DMSO-*d*_6_) δ 8.68 (s, 3H), 7.54 (s, 1H), 7.07 – 6.99 (m, 2H), 6.64 (d, *J* = 8.2 Hz, 1H), 5.57 (br. s, 1H), 5.33 (dt, *J* = 8.1, 6.0 Hz, 1H), 4.60 (br. t, *J* = 5.0 Hz, 1H), 4.51 (qd, *J* = 6.6, 3.3 Hz, 1H), 4.34 (dd, *J* = 8.1, 3.5 Hz, 1H), 3.91 – 3.75 (m, 2H), 2.84 (dd, *J* = 16.1, 6.2 Hz, 1H), 2.74 (dd, *J* = 16.1, 5.9 Hz, 1H), 1.14 (d, *J* = 6.6 Hz, 3H).

**
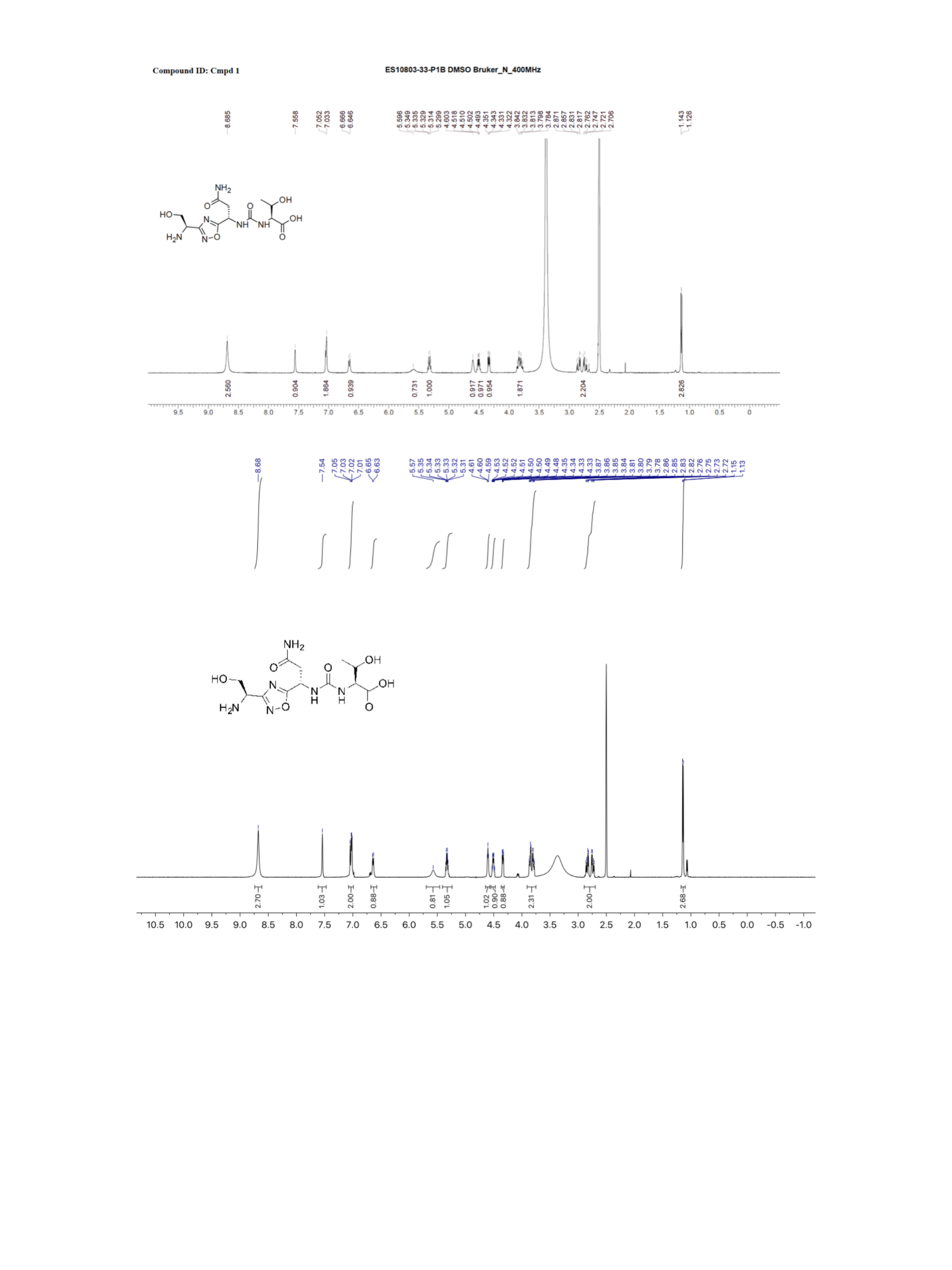
**

**Fig. S11:** ^13^C NMR of Aurigene-1 in DMSO-*d_6_*. ^13^C NMR was run on a Varian 700 MHz spectrometer. ^13^C NMR (175 MHz, DMSO-*d*_6_) δ 181.6, 172.0, 170.4, 166.0, 156.7, 71.3, 60.0, 57.1, 48.8, 44.1, 38.3, 16.4. LRMS (ESI+) m/z calculated for C_12_H_21_N_6_O_7_ [M+H]^+^ 361.1; found 361.2.

**
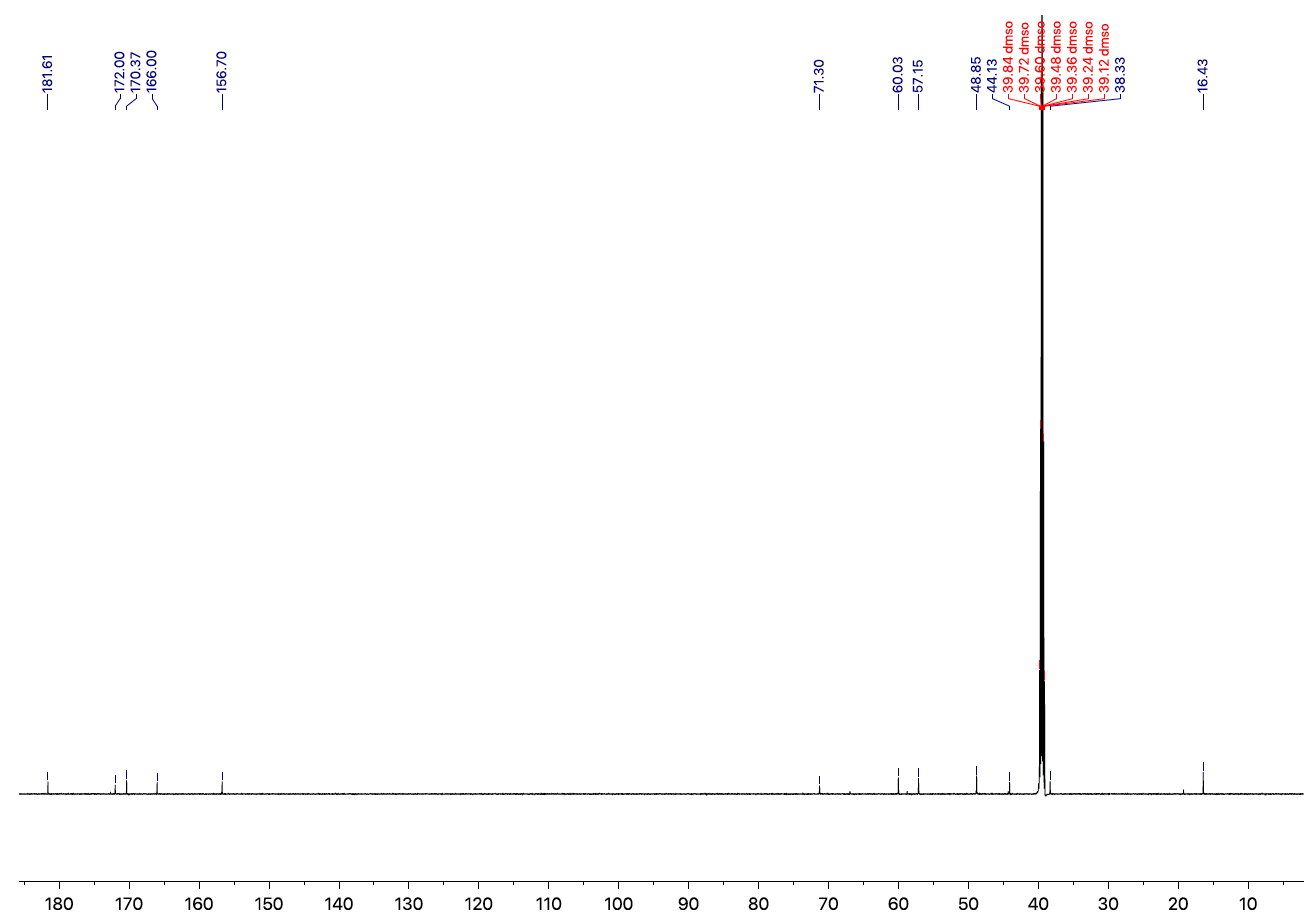
**

**Fig. S12:** Low resolution mass spectrum of Aurigene-1 provided by the supplier.


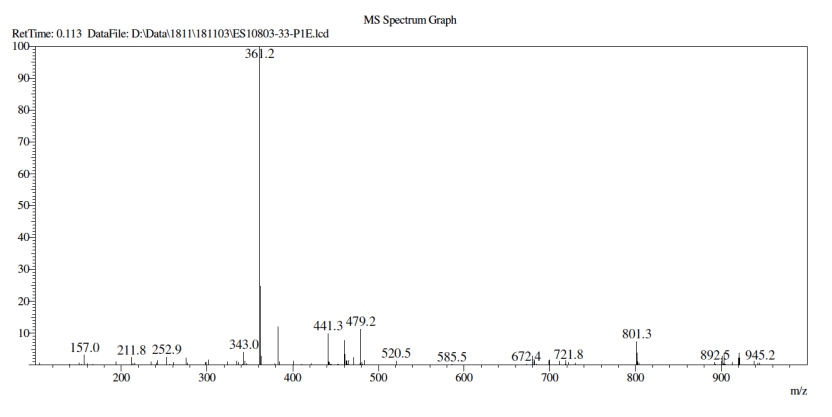


**Fig. S13:** Liquid chromatography-mass spectroscopy (LCMS) analysis of compound A. LC of LCMS for Compound A is provided.


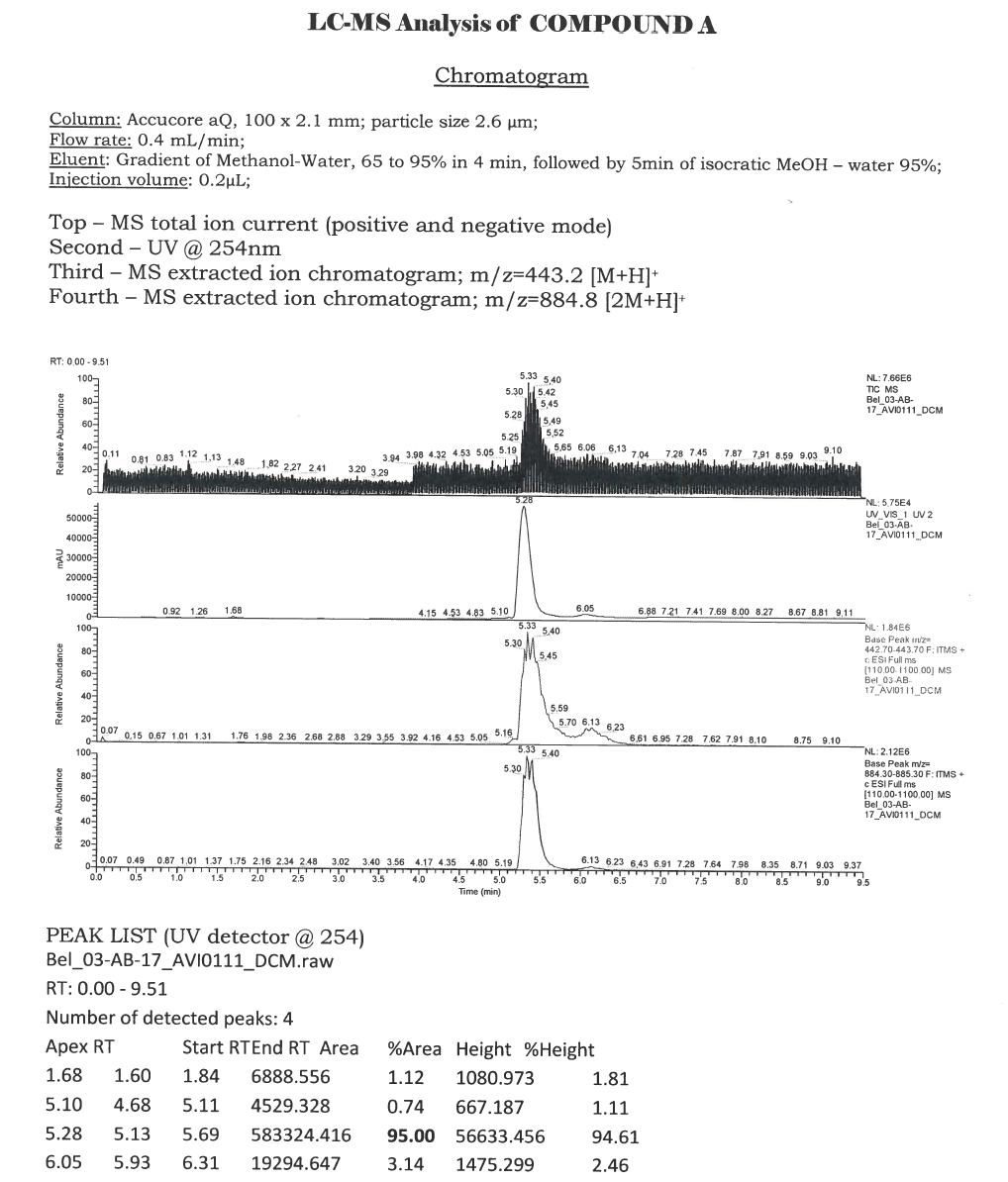


**Fig. S14:** Liquid chromatography-mass spectroscopy (LCMS) analysis of compound A. MS of LCMS for Compound A is provided.


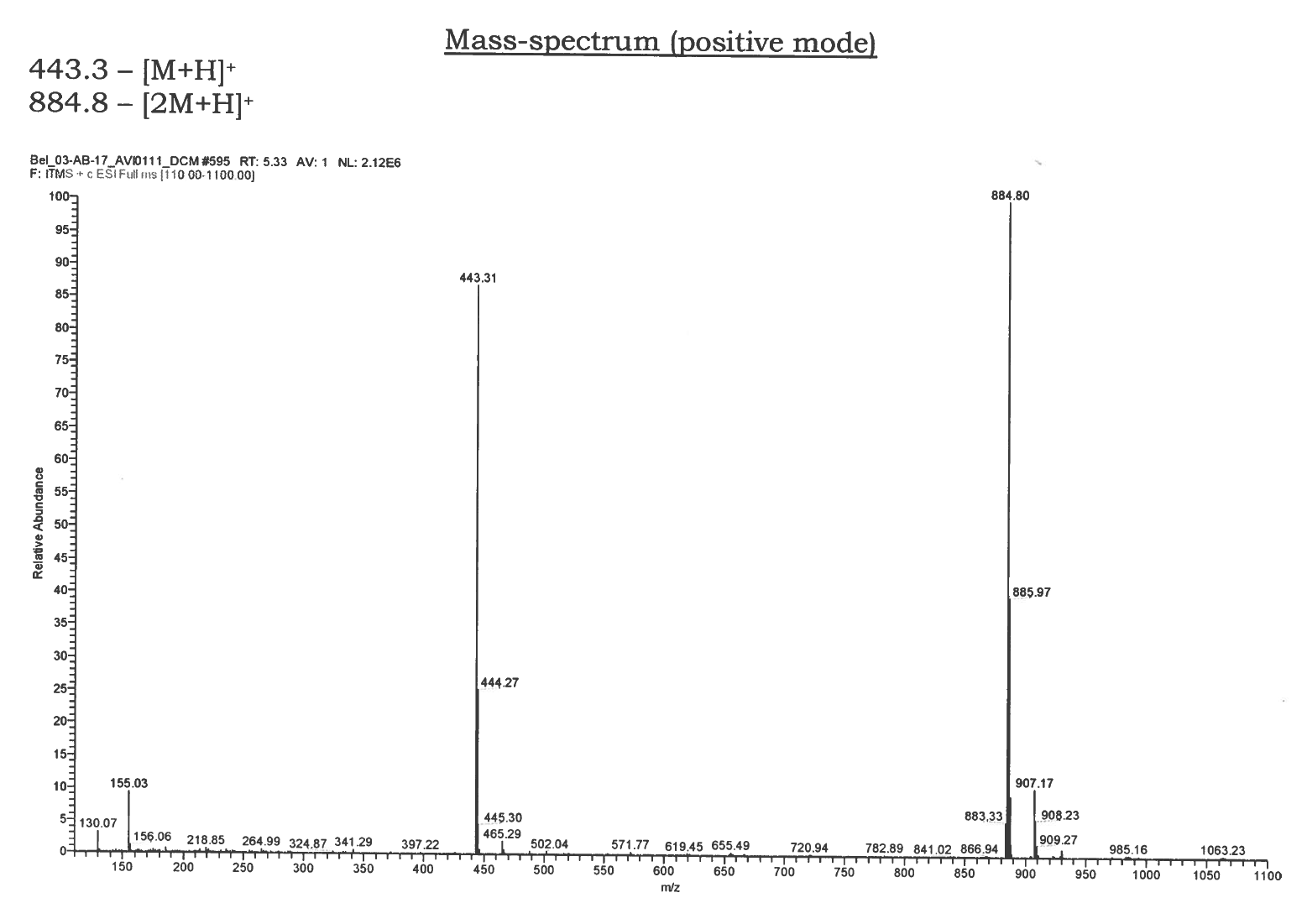


**Fig. S15:** ^1^H NMR spectra of compound A.

**Fig. S16:** ^13^C NMR spectra of compound A.

**
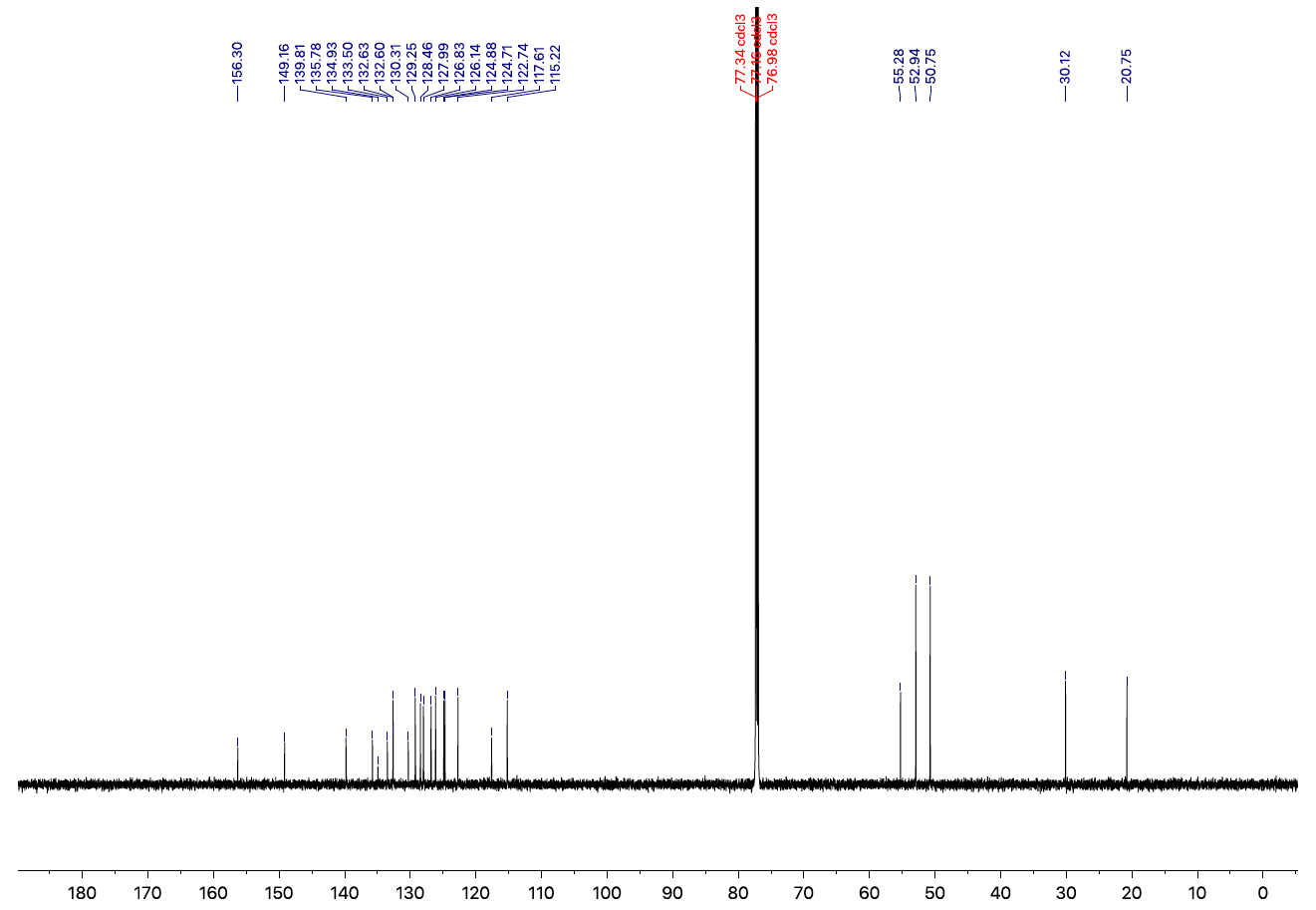
**

**S. Text 1:**

**PD-1:PD-L1 ELISA Competition Assays (reverse configuration)**

High-binding 96 well plates (Corning Inc, Kennebunk, ME) were coated with 0.1 µg/100 µL/well of Fc-tagged human PD-1 (Acro Biosystems). Standard curves were generated in each plate with 0-1,000 ng/mL biotinylated human PD-L1 (Acro Biosystems). Test compounds, peptides or antibody (Acro Biosystems) were diluted in buffer (PBS, 0.5 % BSA, 0.05 % Tween 20, 0.5 % DMSO) then pre-incubated in the coated wells for 30 minutes before adding 150 ng/mL biotinylated human PD-L1. This ligand concentration used for testing compounds was chosen based on the linear range of the standard curve (concentration which fall into > 50% of linear range in the standard curve). After one-hour incubation with the ligand, the plate was washed, and the bound ligand was detected with the addition of streptavidin-HRP (R&D Systems) followed by development with TMB solution (SeraCare Life Sciences Inc, Milford, MA). Binding was quantified in relation to the ΔA_450_ (A_450_ from sample - A_450_ from uncoated background) of the standard curve in each assay plate using a four parameters logistic nonlinear regression (GraphPad Prism v 7.03). Results were presented as inhibition % [{1-(binding of biotinylated ligand in the presence of compounds/binding of biotinylated ligand in the absence of compounds)}x100].

**S. Text 2:**

**Molecular docking calculations**

All docking calculations in this study were carried out using Molecular Operating Environment, MOE 2018.01, package^1^. All proteins used for docking calculations were initially prepared using the MOE protein preparation wizard, during which process any missing loops were constrcted and the protanation state of each of the ionizable residues in the protein structures were assigned using the Protonate 3D algorithm^1^.

The 3D structures of the human PD-L1 complexes with the two BMS small molecules, BMS-103 and BMS-142, have not been experimentally resolved. However, the X-ray crystal structure of PD-L1 bound with BMS-202, which belongs to the same (2-methyl-3-biphenylyl)methanol scaffold as BMS-103 and BMS-142, is available in the Protein Data Bank (PDB: 5J89)^2^. Therefore, the interactions of BMS-202/PD-L1 complex from PDB were used to guide the modelling of the BMS-103/PD-L1 and the BMS-142/PD-L1 complexes. Initially, four important 3D pharmacophore fingerprints of BMS-202, such as three aromatic ring features and a hydrogen-bond (or H-bond) donating amine group, were selected and defined using the Unified Annotation Scheme in MOE program^1^. Later, these pharmacophore features were specified and BMS-103 and BMS-142 were initially docked into the binding site of BMS-202 within a PD-L1 dimer complex using the pharmacophore placement method. Upto 100 poses were generated and scored using the LondonDG scoring function. Subsequently, the poses from initial placement were refined using the induced-fit method, in which the binding site residues within a 6 Å cutoff distance were treated flexibly, and the poses were scored using the AffinitydG method in MOE^1^. The top-most ranking pose for the BMS-103/PD-L1 and the BMS-142/PD-L1 complexes that satisfied all the pre-set pharamacophore requirements were chosen for further molecular dynamics (MD)-based refinment proceduce (as disussed below).

Since there is no information about the binding site and/or binding mode of Aurigene-1 with human PD-L1 protein, we explored the plaussible binding pockets in all the available X-ray crystal structures of human PD-L1 in PDB. For this purpose, we treated all the chains (if present) in the individual PDB complex as independent structures during our docking calculations. For example, the X-ray crystal structure of human PD-L1 in complex with atezolizumab monocolonal antibody (mAb) includes five chains of PD-L1 (namely, A-E). So we included all these five chains as independent target structure during docking. By doing so, we generated an ensemble of 48 PD-L1 structures from PDB, which were prepared using MOE protein preparation protocol. Subsequently, all possible binding pockets in each of the 48 PD-L1 structures were identified using the MOE site finder methodology that is based on alpha spheres^1^ and Aurigene-1 compound was docked and in all the pockets seperately. In this process, the triangle matcher method and LondonDG scoring function were employed to generate 100 poses for each Aurige-1/PD-L1 complex. Later, induced-fit methodology was used to refine each of the complexes within a flexibile environment and scored using the AffinitydG method. This two-step docking stage resulted in a total of 480 Aurigene-1/PD-L1 complexes. Among these poses, only those in which Aurigene-1 bound at the GFCC’C” face (or PD-1 binding face) of PD-L1 IgV domain were selected for further MD refinement and analyses.

**Molecular dynamics and binding affinity calculations**

All the molecular dynamics (MD) simulations were performed using the Amber 14 package and the Amberff14SB force fields^3^. The structures of all the small-molecule/PD-L1 complexes (such as BMS-103/PD-L1, BMS-142/PD-L1, and Aurigene-1/PD-L1) obtained from preliminary docking calculations were solvated in a periodic cubic box of explicit TIP3P water molecules such that no atom in the system was within 12 Å from any side of the box. The solvated systems were further neutralized with sodium and chloride counter-ions using the tleap program in the Amber 14 package^3^. Subsequently, the systems were subjected to 15,000 steps of energy minimization, during which harmonic force restraints of 100 kcal mol^-1^ Å^-2^ (for 5000 steps), 50 kcal mol^-1^ Å^-2^ (for 5000 steps), and 5 kcal mol^-1^ Å^-2^ (for 5000 steps) were applied. The energy-minimized complexes were then heated to 310 K, by placing a 5 kcal mol^-1^ Å^-2^ restraint on the solute atoms. Following heating, each of the systems underwent a short equilibration of 100 ps and a 2 ns long MD production run. All harmonic restraints were removed during the production simulations.

Following the MD simulations, the small-molecule/PD-L1 complexes in this study were rescored using the computed binding free energy scores that were calculated using molecular mechanic-generalized Born surface area (MM-GBSA) approach. For each MD trajectory produced, 10 snapshots collected at a regular interval of 100 ps from the last 1 ns time scale were employed for MM-GBSA calculations. The binding free energy (ΔG_bind_) using the MM-PB(GB)SA can be estimated as,

| $\Delta G_{bind}= \Delta E_{MM}+\Delta G_{Solv}-T\Delta S$ | (1) |
| --- | --- |

Here, Δ*E_MM_* refers to the molecular mechanical energy and it is the sum of all energies from the bonded and non-bonded interactions. The solvation energy, Δ*G_solv_*_,_ is the sum of the polar and non-polar contributions of solvation. The polar solvation terms (Δ*G_PB/GB_*) are estimated using a Generalized-Born model or a Poisson-Boltzmann solver. The non-polar contributions are computed based on the size of the solvent-accessible surface area (Δ*G_SASA_*) in the ligand and protein. The final component of the above equation is TΔ*S*, which corresponds to the conformational entropy changes in the reaction-product (i.e., protein-ligand complex), upon ligand binding. The estimation of entropy contribution is computationally expensive, therefore, this factor was only calculated for the selected complexes, including those Aurigene-1/PD-L1 complexes having > 25 kcal mol^-1^ relative binding free energy (i.e., without TΔ*S*) along with the BMS-103/PD-L1 and the BMS-142/PD-L1 complexes. During the calculation of entropy contributions, only 5 snapshots were used so as to reduce the computational rigorousness. For all the MM-GBSA calculations in this work, we employed the implicit GB solvent model, GB-Neck2 (igb=8)^4^ for calculating the electrostatic solvation-free energy and the salt molar concentration was set to 0.15 M. All MMGBSA analyses in this study were carried out using the MMPBSA.py script included in the AmberTools.

**Reference**

1. Molecular Operating Environment (MOE); 2013.08 (Chemical Computing Group ULC, 1010 Sherbooke St. West, Suite #910, Montreal, QC, Canada, H3A 2R7, 2019).

2. Zak, K. M. *et al.* Structural basis for small molecule targeting of the programmed death ligand 1 (PD-L1). *Oncotarget* **7**, 30323-30335, doi:10.18632/oncotarget.8730 (2016).

3. Maier, J. A. *et al.* ff14SB: Improving the Accuracy of Protein Side Chain and Backbone Parameters from ff99SB. *J. Chem. Theory Comput.* **11**, 3696-3713, doi:10.1021/acs.jctc.5b00255 (2015).

4. Nguyen, H., Roe, D. R. & Simmerling, C. Improved Generalized Born Solvent Model Parameters for Protein Simulations. *J. Chem. Theory Comput.* **9**, 2020-2034, doi:10.1021/ct3010485 (2013).
